# Niclosamide and Palbociclib Act Synergistically to Reduce Cholangiocarcinoma Cell Viability In Vitro and Inhibit Tumour Growth in a Mouse Model

**DOI:** 10.3390/cancers17223721

**Published:** 2025-11-20

**Authors:** Grace Martin, Ka Ying Lee, Christopher Roberts, Jinxia Zheng, Gagan Kaur Batth, William Dalleywater, Farhat Latif Khanim, Sebastian Oltean, Kevin Gaston, Padma-Sheela Jayaraman

**Affiliations:** 1 School of Medicine and Biodiscovery Institute, University of Nottingham, Nottingham NG7 2RD, UK; 2 Institute of Cancer and Genomic Sciences, University of Birmingham, Birmingham B15 2TT, UK; 3 Department of Clinical and Biomedical Sciences, University of Exeter Medical School, Exeter EX1 2LU, UK; 4 School of Biomedical Sciences, University of Birmingham, Birmingham B15 2TT, UK

**Keywords:** bile duct, cholangiocarcinoma, niclosamide, CDK4/6 inhibition, primary liver cancer

## Abstract

**Background:** Despite the emergence of new treatment modalities, including targeted therapies that are of benefit to patients whose tumours carry specific mutations, the prognosis for most patients with cholangiocarcinoma remains poor. Novel therapeutic approaches that can benefit the majority of patients whose tumour cells do not carry targetable mutations are urgently needed. **Results:** To identify mutation-agnostic treatment approaches, we screened a library of well-tolerated off-patent drugs against cholangiocarcinoma cells and normal biliary epithelial cells. The screen identified Niclosamide as a drug that reduces the viability of multiple cholangiocarcinoma cell lines but has a lesser effect on normal primary biliary epithelial cells. Moreover, Niclosamide treatment reduces the growth of cholangiocarcinoma tumour cells as tumour spheroids in vitro and reduces the growth of cholangiocarcinoma cells as tumours in a xenograft mouse model of this disease. Through a proteasome-dependent mechanism, Niclosamide treatment reduces the expression of the Proline-Rich Homeodomain (PRH) protein, a transcription factor which acts as an oncoprotein in cholangiocarcinoma cells. However, PRH knockout does not alter the sensitivity of cholangiocarcinoma cells to Niclosamide, indicating that this drug is not dependent on PRH to reduce cell viability. Interestingly, the CDK4/6 kinase inhibitor Palbociclib selectively reduces the viability of cholangiocarcinoma cell lines compared to normal biliary epithelial cells and, importantly, Palbociclib synergises with Niclosamide to reduce cholangiocarcinoma cell viability in vitro as well as to reduce tumour growth in a mouse xenograft model. **Conclusion:** These preclinical results suggest that the combination of Niclosamide and an inhibitor of CDK4/6 is worthy of clinical evaluation as a potential treatment for this disease.

## Simple Summary

Cancer of the bile duct, or cholangiocarcinoma, is increasing in incidence and mortality. Despite the emergence of new targeted therapies that benefit a minority of patients whose tumours carry specific mutations, the outcomes for most patients are very poor, and new treatment strategies are urgently required. Here we show that in preclinical research cholangiocarcinoma cells are more sensitive to Niclosamide, a drug that is commonly used to treat parasitic infections, than normal bile duct cells. Importantly, the combination of Niclosamide with Palbociclib, a drug that blocks cell division, is significantly more effective than either drug alone. Further studies will be required to determine whether this drug combination is effective in the clinic.

## 1. Introduction

Cholangiocarcinoma (CCA), or cancer of the bile duct, is a malignancy that arises in the biliary system that drains bile into the gall bladder and small intestine. CCA is the second most common primary liver cancer after hepatocellular carcinoma (HCC); however, the incidence of CCA has been steadily rising worldwide [1,2]. Moreover, in England rising incidence of CCA has also been observed, with mortality parallel to incidence [3]. At present, the only curative treatment for CCA is surgical resection. However, less than a third of patients are suitable for resection as the disease is usually diagnosed at a late stage [1]. For patients with advanced CCA, Gemcitabine–Cisplatin plus the PDL1 inhibitor Durvalumab is the current standard of care [4]. Targeted therapies have been shown to be of benefit for some patients whose tumours carry specific mutations [5]: Pemigatinib, a pan-FGFR inhibitor, is of value to patients with FGFR2 gene rearrangements [6]; Larotrectinib and Entrectinib, neurotrophic receptor tyrosine kinases (NTRK) inhibitors, are effective in patients with NTRK fusion-positive cancers [7]; Pertuzumab plus Trastuzumab is useful for patients with HER2 amplification [8]; and Ivosidenib is beneficial to patients with IDH1 mutated CCA [9]. However, most patients do not have tumours that carry one of these actionable mutations, and new treatments that benefit all CCA patients would be a great clinical benefit.

Genetic mutations are not the only drivers of the cancer phenotype. The over-expression of a variety of proto-oncogenes can drive tumourigenesis or tumour progression, either with or without, gene amplification. The Proline-Rich Homeodomain (PRH) protein [10], also known by its gene name haematopoietically expressed homeobox (HHEX) HHex protein, is a DNA binding transcription factor that is essential for the formation of multiple tissues [11,12,13], including both liver and bile duct [14], and its expression is regulated by bile acids in the liver [15]. PRH is not expressed at high levels in normal bile duct epithelial cells, but in CCA cells, PRH is highly expressed, and this increases cell proliferation through the regulation of genes controlling the cell cycle and the Wnt and Notch signalling pathways [16]. Moreover, knockdown of PRH gene expression in CCA cells reduces the formation and growth of xenograft tumours in nude mice [16]. Over-expression of PRH is also important in several other solid tumours and in some lymphomas and leukaemias [12,13]. In contrast, in multiple cell types, including prostate and breast epithelial cells, PRH negatively regulates cell proliferation, and PRH expression is decreased or lost in tumours [17,18]. This suggests that PRH is a member of a group of transcription factors and kinases that act as proto-oncogenes with tumour suppressor function [19].

CCA cells over-expressing the PRH protein have a higher rate of cell proliferation compared to control cells, and this increases the sensitivity of these cells to the CDK4/6 inhibitor Palbociclib [16]. In an effort to identify other small molecules that inhibit the proliferation of CCA cells, we report here the results obtained in a screen using a repurposing library of off-patent drugs that we have described previously [20]. The screen identified Niclosamide as a potent inhibitor of CCA cell viability. Niclosamide is a salicylic acid derivative originally identified as an anti-helminth agent and subsequently shown to decrease the viability of multiple cancer cell types [21,22], including HCC cells and CCA cells [23,24]. Niclosamide has long been known to depolarise mitochondrial membrane potential to uncouple electron transfer [25]. In addition, in cell lines from a variety of different tissues, Niclosamide can promote apoptosis and autophagy and inhibit many signalling pathways, including STAT-3 phosphorylation, NF-kB activity Notch, and Wnt [21,22]. Here we show that Niclosamide promotes global protein ubiquitination levels, decreases PRH protein levels in CCA cells, and reduces the growth of CCA tumours in a xenograft mouse model. Niclosamide inhibits cell cycle progression in CCA cells through a reduction in cyclin D1 and β-catenin expression and a decrease in Rb phosphorylation and also promotes apoptosis. Interestingly, the presence of PRH is not essential for the inhibitory effects of Niclosamide on CCA cells, confirming that the effects of this drug are mediated by many factors. More importantly, Niclosamide and Palbociclib act synergistically to decrease CCA cell viability in vitro as well as act synergistically to inhibit tumour growth in a mouse xenograft model.

## 2. Materials and Methods

### 2.1. Chemicals

Palbociclib (SelleckChem, S4482 Houston, TX USA) was solubilised in DMSO in Eppendorf tubes by vortexing violently for two minutes and then sonicated (Transsonic T310, Camlab, Cambridge, UK) in a 50 °C water bath. If precipitate was present, the warming and sonication continued until no precipitate remained. Stocks at 10 mM and 1 mM were stored at −20 °C for short-term use (3 months) and −80 °C for longer-term use. Niclosamide (Merck-Millipore, 481909-1GM, Burlington MA, USA) was dissolved in DMSO to make 10 mM and 1 mM stocks and stored as above. Protein stability experiments were conducted using the proteasome inhibitor, MG132 (Sigma Aldrich, M7449, Burlington, MA, USA).

### 2.2. Cell Culture

CCLP, RBE, and KKU-M055 CCA lines were a gift from Professor David Bates (University of Nottingham UK), and their identity was confirmed by STR profiling. The CCA cell lines have different genetic backgrounds and aetiology. KKU-M055 were derived from a patient with liver-fluke infection and carry a MAP2K1 (K57T) mutation, whereas CCLP and RBE cells are not liver-fluke–related. CCLP cells express mutant TP53 while RBE cells express KRAS (G12V) and IDH1 (R132S). CCA cell lines and modified derivatives were cultured in Dulbecco’s Modified Eagle Medium (DMEM, D5796 Sigma), with 10% foetal bovine serum (FBS, F7524, Sigma) at 37 °C and 5% CO_2_ in a humidified chamber for a maximum of 30 passages. Primary Biliary Epithelial Cells (BECs) obtained from healthy human liver tissue (Innoprot, Elexalde Derio, Spain) were grown in Epithelial Cell Media (Innoprot, P60106, Elexalde Derio, Spain) supplemented with 2% FBS and 1% Epithelial Growth Supplement, as per the supplier’s protocol for a maximum of ten passages. All cells were tested monthly for mycoplasma. Spheroids were generated by seeding 1 × 10^3^ cells per well in 200 μL complete media with 100 μg/mL Matrigel Basement Membrane Matrix (356234, Corning, New York, NY, USA) in round-bottomed, ultra-low-attachment 96-well plates (7007, Fisher Scientific, Waltham, MA, USA) centrifuged at 300× *g* for 10 min and then grown at 37 °C and 5% CO_2_ in a humidified incubator for 7 days. The spheroids were imaged using a Nikon Plate Reading Widefield Microscope at X4 magnification (Nikon, Nishi Oi, Japan), and the images were analysed using ImageJ (1.54m).

### 2.3. Generation of PRH Knockout CCLP Cells

CCLP clones without detectable expression of PRH protein (‘PRH knockout’) were generated using the Cas9 nickase (Cas9n) system with pairs of single-guide RNAs (sgRNAs) targeted against Exon 1 and Exon 2 of the *HHEX* gene. Pairs of spacer sequences targeting *HHEX* Exon 1 and Exon 2 were designed using Benchling (Table 1). The sgRNAs for Exon 1 target either side of the ATG start codon of PRH, in a “PAM-out” orientation, with a distance between nick sites (“nick distance”) of 57 bp. The sgRNAs for Exon 2 target the centre and 3′ end of Exon 2, in a “PAM-out” orientation, with a nick distance of 98 bp. Oligonucleotide sequences corresponding to these spacer sequences were synthesised (Merck), annealed, and ligated into the sgRNA backbone segment of the plasmid pSpCas9n(D10A)-2A-GFP (‘pX461′ a gift from Dr Feng Zhang; Addgene plasmid #48140) to produce four separate plasmids, each encoding Cas9n(D10A)-2A-GFP and one of four sgRNAs targeting *HHEX* Exon 1 or Exon 2 (Table 2). The sequences of encoded sgRNAs in each plasmid were confirmed by Sanger sequencing (Source Bioscience, Cambridge, UK).

CCLP cells were co-transfected using Lipofectamine 3000 (Invitrogen, Carlsbad, CA, USA) according to the manufacturer’s instructions. After 48 h, the cells were subjected to fluorescent activated cell sorting (FACS) based on GFP expression (University of Nottingham Flow Cytometry Unit). Cells were sorted to a density of a single cell per well of a 96-well plate and cultured for 1 month, with regular checking for colony growth. Once each colony had reached sufficient size, it was passaged and expanded.

### 2.4. Western Blotting

The following primary antibodies were used for Western blotting: β-actin 4970S Cell Signalling, β-catenin 8480S Cell Signalling, Cleaved Caspase 3 MAB835, Bio-Techne, Cyclin D1 2978S Cell Signalling, Cyclin D2 3741S Cell Signalling (Danvers, MA, USA), PRH/HHEX 2018B FAB83771C Bio-Techne, P21 2947S Cell Signalling, Phospho-RB 8516S Cell Signalling, Vimentin 5741S Cell Signalling, and Ubiquitin 3936S, Cell Signalling, (Danvers, MA, USA).

### 2.5. Quantitative RT-PCR

Total RNA was extracted using a Bioline Isolate II kit according to the manufacturer’s protocol and reverse-transcribed (QuantiTect Reverse Transcription kit (Qiagen, 205311, Venlo, The Netherlands)). qRT-PCR was performed using a Rotor-Gene Q cycler (Qiagen, Venlo, The Netherlands) and a QuantiTect SYBR green PCR kit (Qiagen, 204143, Venlo, The Netherlands). The expression of genes of interest was normalised to β-actin mRNA using the Pfaffl method [26]. The primers used were β-actin forward 5′-AAAGACCTGTACGCCAACAC-3′, reverse 5′-GTCATACTCCTGCTTGCTGAT-3′, HHEX forward 5′-AAACCTCTACTCTGGAGCCC-3′, and reverse 5′-GGTCTGGTCGTTGGAGAATC-3′.

### 2.6. Cell Viability and Mitochondrial Activity Assays

Cells were plated 1 × 10^4^ per well in 96-well plates and left to adhere for 12 h before drug treatment. Cell viability was then measured using an MTT assay (Merck (M5655-1G) according to the supplier’s instructions in complete media. To examine the viability of cells grown as spheroids, spheroids were grown for 7 days and before imaging using a Nikon Plate Reading Widefield Microscope at X4 magnification and beginning drug treatment. Cell viability was then measured over time using a PrestoBlue assay (A13261, Invitrogen) with a FluorSTAR 96-well plate reader at wavelengths 544 nm (Excitation) and 590 nm (Emission) and gain 750. Images were also taken using a Nikon Plate Reading Widefield Microscope at X4 magnification. Images were analysed using ImageJ. Mitochondrial activity was measured using MitoTracker^®^ Red FM (Invitrogen, Carlsbad, CA, USA). Cells were plated at 5 × 10^4^ per well in a 24-well plate as above and then incubated with media containing 200 nM MitoTracker at 37 °C for 1 h. The cells were then imaged using a Nikon live microscope (Nikon, Nishi Oi, Japan) (570 nm excitation) at 20× magnification, and the images were analysed using ImageJ (1.54m). Mitochondrial staining intensity was determined in 30–50 cells per image, and the intensity value was normalised to vehicle-treated cells.

### 2.7. Drug Dose Response Curves

Cell viability was determined using an MTT assay as above and after subtracting a background control (wells containing 100% DMSO-killed cells). Values were normalised to vehicle control and plotted in GraphPad Prism (10.6.1) to generate dose–response curves. EC50 values were calculated through the analysis tool: non-linear regression (curve fit), dose–response inhibition, log(inhibitor) vs. response (three parameters).

### 2.8. Drug Synergism Calculations

We used the Chou–Talalay dose–effect–based approach, which accounts for the amount of each drug needed to achieve a response as single agents and in combination, and produces a combination index (CI) value as a quantitative measure of synergism or antagonism [27]. The raw data was used to calculate a line of best fit between the log of the drug concentration (logD) and the log of the response (log(Fa/Fu)). The Fraction affected (Fa) and the Fraction unaffected (Fu) were taken from the MTT cell viability data to draw Median Effect plots and produce the CI.

### 2.9. Flow Cytometry

To examine cell cycle progression, fixed cells were treated with RNase (PureLink RNase A (2201416, Invitrogen) and then stained with Propidium Iodide (P4864, Sigma) before analysis using a CytoFlex Station Flow Cytometer. A FITC Annexin V Apoptosis Detection kit (556547, BD Pharmingen TM, San Jose, CA, USA) was used to examine cell death. PI (Excitation 535 nm Emission 610 nm) and Annexin V-FITC (Excitation 488 nm Emission 519 nm).

### 2.10. Mouse Xenograft Model

CCLP cells were grown in vitro, and then 2 × 10^6^ cells were injected subcutaneously into CD1 nude mice. Tumours were grown in the mice until they reached 3 mm in diameter. Palbociclib at 10 mg/kg, Niclosamide at 20 mg/kg, vehicle control (2% DMSO in PBS), or Palbociclib plus Niclosamide were then administered three times weekly intraperitoneally for 30 days. The effect of treatment on tumour volume was measured using callipers over 30 days or until tumours reached the maximum allowed diameter of 12 mm when the mice were culled. Tumour volume was calculated using the formula: [(length + width)/2] × length × width.

### 2.11. Histology and Immunohistochemistry

Xenograft FFPE tissue sections were stained using Harris’s haematoxylin (ThermoFisher Scientific; Waltham, MA, USA) and bluing reagent (ThermoFisher Scientific) and eosin, and mounted with coverslips using Shandon Slide Mountant Media (ThermoFisher Scientific; Waltham, MA, USA). For immunohistochemistry, antigen retrieval was performed by incubating rehydrated slides at 95 °C for 20 min submerged in sodium citrate buffer (pH 6). Immunohistochemistry was then performed using the Shandon Sequenza Immunostaining System (ThermoFisher Scientific). Apoptotic cells were identified by staining for cleaved caspase-3 (cleaved Caspase 3 antibody Asp175, 8 µg/mL (R&D systems, Minneapolis, MN, USA)) using an immunohistochemistry kit (Mouse and Rabbit Specific HRP/DAB IHC Detection Kit-Micro-polymer; Abcam, Cambridge, UK) according to manufacturer’s instructions. After incubating the slides in Mayer’s haematoxylin for 2 min, followed by bluing reagent they were mounted as above. Stained slides were scanned at 20× resolution using a Ventana DP200 slide scanning system (Roche; Basel, Switzerland), using routine slide scanning settings, and saved in TIF format. Scanned images were loaded into QuPath (QuPath-0.5.1-arm64) for analysis and images were exported at low (2×) and high (20×) magnification to demonstrate areas of interest.

### 2.12. Statistical Analysis

Statistically analyses were performed using Microsoft Excel or GraphPad Prism. An unpaired *t*-test was used to assess statistically significant differences, unless stated otherwise, and experiments repeated to N = 3 biological repeats were declared significant if *p*-value < 0.05. Error bars represent standard error of the mean (SEM), unless stated otherwise.

## 3. Results

### 3.1. Drug Screening with a Repurposing Library Identifies Niclosamide as a Potent Inhibitor of CCA Cell Viability

**Figure 1:**
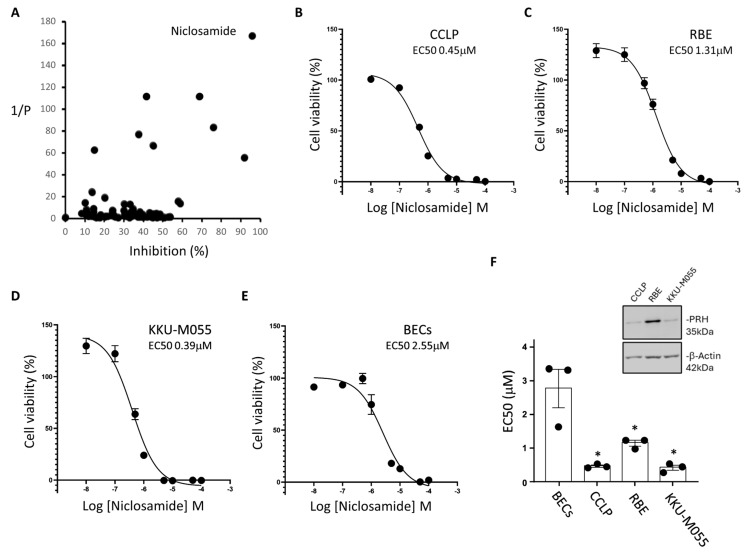
Drug screening and validation of Niclosamide using CCA cell lines and primary biliary epithelial cells. (**A**) A library of 104 off-patent drugs was screened using the CCLP CCA cell line. CCLP cells were plated at 1 × 10^4^ cells per well in a 96 well plate and treated with each drug at its clinically relevant peak serum concentration for 96 h. Cell viability was then determined using an MTT assay and normalised to cells treated with the corresponding vehicle control for each drug. N = 3 biological repeats with a paired, two-tailed T-test followed by Benjamini–Hochberg multiple corrections. The inhibition of viability (%) is plotted against the reciprocal of the *p* value for each drug and Niclosamide is labelled. (**B**–**E**) Niclosamide dose–response curves for CCA cell lines (CCLP, RBE, and KKU-M055) and primary biliary epithelial cells (BECs) plated as in (**A**) and treated with Niclosamide [10 nM–100 μM] or vehicle control for 72 h. Cell viability was measured using an MTT assay and normalised to vehicle control. N = 3 biological repeats each performed in triplicate. Error bars represent SEM. When error bars cannot be seen they are smaller than the symbols. (**F**) The graph shows the relative EC50 values for each cell type calculated using GraphPad Prism. Error bars represent SEM (* = *p* < 0.05 unpaired *t*-test). The inserted Western blot shows PRH levels in the CCA cell lines prior to treatment and with β-actin as a loading control.

We performed a blinded screen using a drug repurposing library of 104 compounds (listed in Table S1) and a CCA cell line. CCLP CCA cells were exposed to the drug library for 96 h using the maximum peak serum concentrations achieved for each compound as reported in the literature following use for standard clinical indications [20]. Cell viability was then measured using an MTT assay and normalised to the vehicle control for each of the 104 drugs. The experiment was repeated three times independently and statistical analysis was carried out using a paired, two-tailed *t*-test (Figure S1A). The anti-helminthic drug Niclosamide had the greatest inhibitory effect on cell viability and the lowest *p* value (Figure 1A).

To determine whether Niclosamide has the same effect against other CCA cell lines we performed dose response experiments using RBE and KKU-M055 CCA cells as well as CCLP cells and normal primary biliary epithelial cells (BECs). All three CCA cell lines showed less viability in the presence of Niclosamide with EC50 values ranging from 0.39 to 1.31 μM (Figure 1B–D and Table 1). Normal BECs also showed reduced cell viability in the presence of Niclosamide under these conditions (Figure 1E). However, a comparison of the EC50 values obtained using BECs and CCA cell lines showed that the normal BECs are less sensitive to Niclosamide treatment (Figure 1F).

To understand the effects of Niclosamide on these cell lines in more detail we measured cell viability after a 24 h incubation with the drug (Figure S1B). In all three CCA cell lines and in BECs, incubation with Niclosamide for 24 h inhibited cell viability and in this case the EC50 values are similar in each case (Table 3). These data suggest that Niclosamide treatment has an initial toxicity that is not cancer cell specific but that at longer time points there is a reduction in cell viability that is more pronounced in the cancer cells. As expected, Niclosamide treatment depolarised mitochondrial membrane potential in all three cells lines indicating that electron transfer is uncoupled (Figure S1C).

### 3.2. Niclosamide Treatment Induces the Degradation of PRH and Decreases the Expression of Multiple Proteins That Control Cell Proliferation

**Figure 2:**
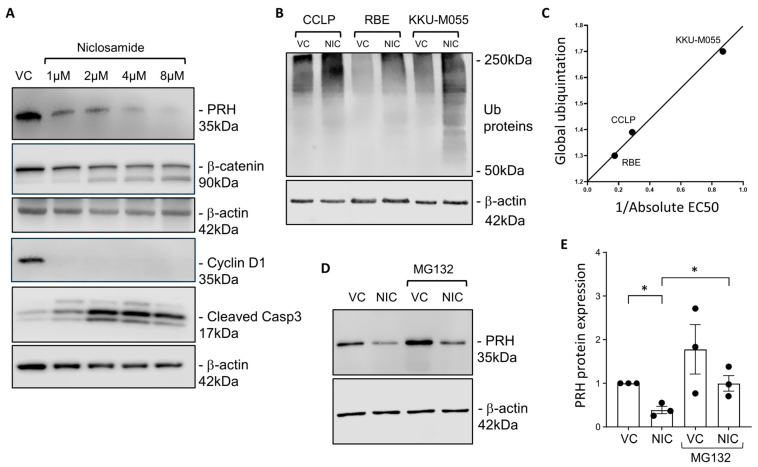
Niclosamide treatment decreases the levels of multiple proteins that determine CCA cell viability. (**A**) CCLP cells were treated with vehicle control (VC) or Niclosamide (NIC) at the concentrations given in the figure for 24 h before harvesting for Western blot analysis using PRH, β-catenin, Cyclin D1 and cleaved Caspase 3 antibodies with β-actin as a loading control. (**B**) CCLP, RBE and KKU-M055 cells were treated with VC or 8 μM Niclosamide (NIC) for 24 h before harvesting for Western blot analysis using a ubiquitin antibody to measure global protein ubiquitination levels with β-actin as a loading control. (**C**) Global protein ubiquitination was quantified by normalising the ubiquitinated proteins to β-actin and relativising to VC and is plotted against the absolute EC50 values observed in each cell line. (**D**) CCLP cells were treated with VC or 8 μM Niclosamide (NIC), as in (**B**) in the absence and presence of 5 μM MG132 and PRH protein levels were determined using Western blot as in (**A**). (**E**) The experiment show in (**D**) was performed to N = 3 and PRH levels relative to VC were determined by Western blot. Error bars represent SEM and * = *p* < 0.05 unpaired *t*-test. Original western blots are presented in File S1.

To examine the effects of Niclosamide on CCA cells in more detail we next used Western blot to assess the effects of this drug on the expression of proteins that are known to be important in the control of CCA cell proliferation. PRH has previously been shown to act as an oncoprotein in CCA cells [16]. Western blotting confirmed that this protein is expressed in the three CCA cells lines used in this study, although, PRH expression levels in these cell lines did not correlate with sensitivity to Niclosamide (Figure 1F). However, treatment of CCLP cells with Niclosamide resulted in decreased expression of PRH (Figure 2A and Figure S2A). Niclosamide treatment also decreased the expression of PRH in RBE cells and KKU-M055 cells (Figure S3). Niclosamide treatment also resulted in decreased expression of β catenin and Cyclin D1 in CCLP cells (Figure 2A and Figure S3) and in RBE and KKU-M055 cells (Figure S3). Niclosamide treatment increased the levels of the apoptosis marker cleaved caspase 3 in CCLP and KKU-M055 cells suggesting that apoptosis is induced in these cells (Figure 2A, Figure S2, Figure S3, respectively). Interestingly, however, cleaved caspase 3 was not detectable in RBE cells suggesting that the effects of Niclosamide on cell viability are not dependent on the presence of caspase 3.

The decreased expression levels of PRH, β-catenin and Cyclin D1 in Niclosamide treated cells suggests that protein turnover is affected in these cells. Consistent with this Niclosamide has been reported to increase protein ubiquitination levels in different cancer cell lines [28,29]. To examine global protein ubiquitination in CCA cells, we performed Western blotting with a pan ubiquitin antibody before and after Niclosamide treatment (Figure 2B). In all three CCA cell lines Niclosamide treatment increased global protein ubiquitination levels (Figure 2B and Figure S4) although the increase in RBE cells was not statistically significant. Interestingly the increase in protein ubiquitination appeared to correlate with the EC50 for Niclosamide in each cell line (Figure 2C). This suggests that sensitivity to Niclosamide is related to the ability of this drug to increase protein ubiquitination.

### 3.3. PRH Is Not Essential for the Effects of Niclosamide on CCA Cell Lines

PRH has been shown to interact with the proteasome [30], and this protein is known to be proteasomally processed in other cancer cell types and to alter cell survival [31]. Since PRH protein levels were down-regulated by Niclosamide in all of the CCA cell lines tested, we next assessed whether PRH is targeted for degradation by the proteasome following Niclosamide treatment. We first confirmed that PRH mRNA levels are not altered by Niclosamide treatment in any of the cell lines (Figure S5A). CCLP cells were then treated with vehicle control or Niclosamide in the absence and presence of the protease inhibitor MG132 and PRH protein levels were determined using Western blot. As expected, PRH proteins levels were decreased by Niclosamide treatment (Figure 2D). Interestingly, MG132 treatment increased PRH levels in the absence of Niclosamide and partially abrogated the down-regulation of PRH protein levels seen in the presence of Niclosamide (Figure 2E). The same results were obtained in RBE cells (Figure S5B,C). These data indicate that PRH is processed or degraded by the proteasome in CCA cell lines and that PRH processing or degradation is induced by Niclosamide.

To determine whether the decrease in PRH protein levels seen on treatment with Niclosamide is essential for the effects of Niclosamide on the CCA cells we made use of CCLP cells in which PRH expression has been knocked out using CRISPR targeting. Full details on the effects of PRH knockout on CCLP cells will be reported elsewhere. However, here we show that the *HHEX* knockout CCLP cells do not express the PRH protein (Figure S6A). Treatment of PRH knockout cells and control cells with Niclosamide did not show any difference in the EC50 for Niclosamide (Figure S6B,C). This shows that the decrease in PRH expression caused by Niclosamide treatment is not solely responsible for the effects of Niclosamide on these cells although it may be a contributory factor. This is consistent with the effects of Niclosamide treatment on global levels of protein ubiquitination and protein expression shown in Figure 2 as well as its effects on mitochondrial membrane potential shown in Figure S1C.

### 3.4. Niclosamide and Palbociclib Act Synergistically in Multiple CCA Cell Lines

**Figure 3:**
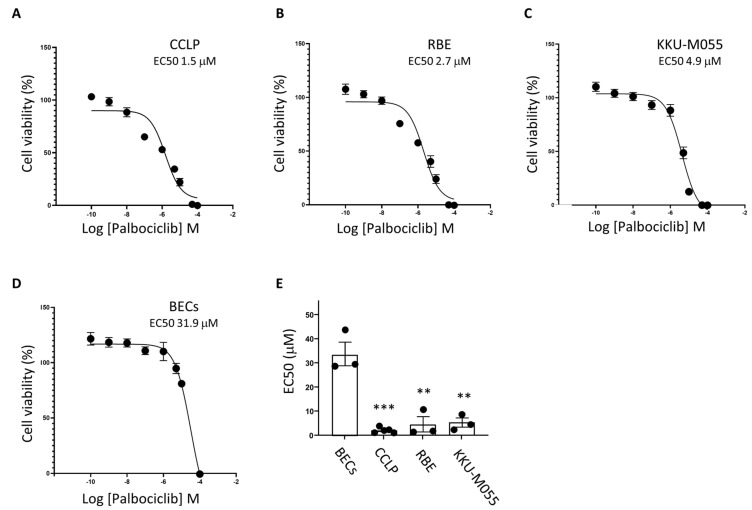
Palbociclib decreases the viability of CCA cells but has less effect on primary biliary epithelial cells. CCA cell lines (CCLP (**A**), RBE (**B**), KKU-M055 (**C**), or primary BECs (**D**)) were plated at 1 × 10^4^ cells per well in a 96-well plate and treated with increasing concentrations of Palbociclib for 96 h. Cell viability was then measured using an MTT assay and normalised to the vehicle control. Three biological repeats (five for CCLP cells) each performed in triplicate. Error bars represent SEM. When error bars cannot be seen, they are smaller than the symbols. (**E**) The graph shows the relative EC50 values calculated using GraphPad Prism. Error bars represent SEM (** = *p* < 0.01 *** = *p* < 0.001 unpaired *t*-test).

Niclosamide acts as a mitochondrial uncoupler and an inducer of apoptosis and it impacts a variety of cell signalling pathways. We therefore considered whether it might act synergistically with other drugs that act by different mechanisms and thus achieve a more potent cell killing effect. Dysregulation of genes encoding cell cycle components is frequent in CCA [32,33] and we have previously demonstrated that cell cycle inhibition using the CDK4/6 inhibitor Palbociclib reduces the viability of CCLP cells [16]. We therefore tested the ability of Niclosamide to synergise with Palbociclib. First, we examined the effect of Palbociclib treatment on the cell lines used in this study and on primary BECs. Palbociclib treatment had a significant inhibitory effect on the viability of CCLP cells, RBE cells and KKU-M055 cells (Figure 3A–C). More interestingly Palbociclib treatment had very little effect on BECs under the same conditions (Figure 3D,E). This differential effect on the cancer cells was also apparent with a shorter incubation with the drug (Figure S7).

**Figure 4:**
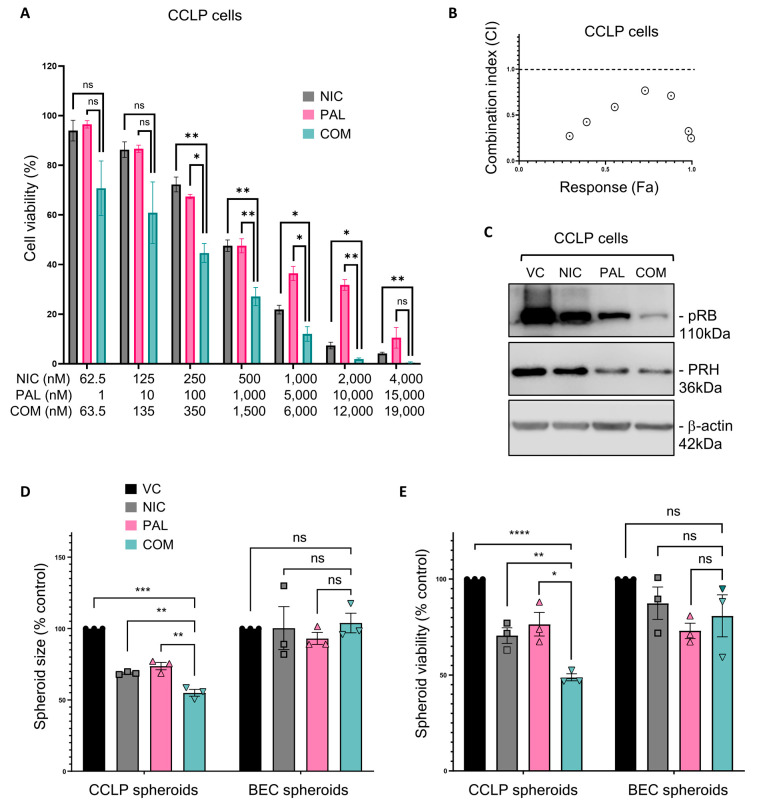
Niclosamide and Pablociclib act synergistically to reduce CCA cell viability. (**A**) CCLP cells were plated at 1 × 10^4^ cells per well in a 96-well plate before treatment with Niclosamide (NIC), Palbociclib (PAL), or both drugs in combination (COM) at the concentrations shown and for 72 h. Cell viability was then measured using an MTT assay. N = 3 biological experiments each performed in triplicate. Error bars represent SEM (* = *p* < 0.05, ** = *p* < 0.01, ns = not significant) two-tailed paired *t*-test. (**B**) From the data shown in (**A**) combination index (CI), values were calculated and plotted against the corresponding response (Fa) values in a CI plot. CI < 1 indicates synergism. (**C**) CCLP cells were treated with 0.5 μM Niclosamide (NIC), 1 μM Palbociclib (PAL), or both drugs in combination (COM) for 24 h, then proteins were extracted for Western blot. (**D**) CCLP cells and BECs were grown as spheroids for 5 days before treatment with 0.5 μM Niclosamide, 1 μM Palbociclib, or both drugs in combination for a further 72 h. Images were taken using a Nikon brightfield microscope, and spheroid size was calculated using ImageJ. N = 3 biological repeats. Error bars represent SEM (* = *p* < 0.05, ** = *p* < 0.01, *** = *p* < 0.001, **** = *p* < 0.0001) two-tailed paired *t*-test. (**E**) Cell viability was measured in the spheroids from (**D**) using a PrestoBlue assay. Original western blots are presented in File S1.

Having shown that both Niclosamide and Palbociclib are more potent against CCA cell lines than BECs, we next tested whether these drugs act in synergy. Drug combination studies were performed as described above using MTT cell viability assays and synergism was assessed using the Chou-Talalay method to generate combination index (CI) values [27]. Increasing the dose of either drug alone or the combination of both drugs were tested in CCLP cells (Figure 4A). As expected, both drugs alone reduced the viability of CCLP cells, and the drugs in combination were more effective than single treatments (Figure 4A). Combination index (CI) values were calculated from these data and plotted against the corresponding response (Fa) values in a CI plot (Figure 4B). The CI values for the two drugs in combination were all <1, indicating synergistic behaviour (Figure 4B). Similar results were obtained using RBE cells (Figure S8A,B) and KKU-M055 cells (Figure S8D,E) although the synergism in KKU-M055 cells was less robust. As might be expected, given that CDK4/6 phosphorylates the RB protein, treatment with Palbociclib reduced the levels of phosphorylated RB (pRB) in the treated cells (Figure 4C). More interestingly, Niclosamide treatment also reduced pRB levels in these cells (Figure 4C), and the combination of Palbociclib and Niclosamide further reduced pRB levels (Figure 4C), suggesting a synergistic effect (quantified in Figure S9A). The same results were obtained in both RBE cells (Figure S8C) and KKU-M055 cells (Figure S8F), adding further weight to the argument for a synergistic effect of the drug combination. Interestingly, both Palbociclib and Niclosamide also reduced the expression of PRH in all three CCA cell lines (Figure 4C and Figure S8C,F).

To determine whether the combination of Niclosamide and Palbociclib has a more potent effect on tumour cells than on BECs under conditions that are more representative of the 3D arrangement of cells found in tissues, we produced CCLP and BEC spheroids and treated them with each drug alone and in combination. CCLP cells and BECs were grown in non-adherent spheroid conditions for 5 days, and the resulting spheroids were then incubated with vehicle, each drug alone, or in combination for a further 3 days. Treatment with Niclosamide alone or Palbociclib alone resulted in a reduction in the size of CCLP spheroids and the number of viable cells but had no effect on the size of BEC spheroids or the number of viable cells (Figure 4D and Figure 4E, respectively). Moreover, we found that the combination of Niclosamide and Palbociclib had a more potent effect on CCLP spheroid size and cell viability than individual treatment but had no effect on the size of BEC spheroids and little or no effect on the viability of BEC cells under these conditions (Figure 4D and Figure 4E, respectively).

To probe the mechanism underlying the observed synergy, Niclosamide and Palbociclib were replaced by an alternative member of their drug class. Replacement of Palbociclib with the alternative CDK4/6 inhibitor Ribociclib also resulted in a synergistic effect when combined with Niclosamide (Figure S10A). Replacement of Niclosamide with an alternative mitochondrial uncoupler, Carbonyl cyanide-4 (trifluoromethoxy) phenylhydrazone (FCCP), also resulted in a synergistic effect when combined with Palbociclib (Figure S10C). These substitution experiments suggest that cell cycle inhibition together with mitochondrial membrane depolarisation are key determinants of synergy between Palbociclib and Niclosamide in these cells.

### 3.5. Niclosamide and Palbociclib Combination Treatment Induces Cell Cycle Arrest and Apoptosis in CCA Cell Lines

**Figure 5:**
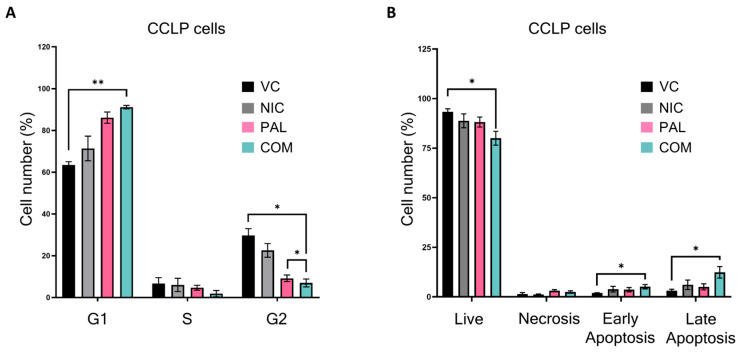
Combining Niclosamide and Pablociclib results in decreased cells in G2 and increased apoptosis. (**A**) CCLP cells were treated with vehicle control (VC), 0.5 μM Niclosamide (NIC), 1 μM Palbociclib (PAL), or both drugs (COM) for 72 h before harvesting for cell cycle analysis using flow cytometry. The graph shows the proportion of the cell population in each stage of the cell cycle. N = 3 biological experiments. Error bars represent SEM (* = *p* < 0.05, ** = *p* < 0.01) two-tailed paired *t*-test. (**B**) CCLP cells were treated with Niclosamide (NIC), Palbociclib (PAL), or both drugs (COM) as in (**A**), then the number of live cells, cells in necrosis, and cells in early and late apoptosis was determined using flow cytometry. Error bars represent SEM (* = *p* < 0.05, ** = *p* < 0.01) two-tailed paired *t*-test.

To understand the mechanisms through which Niclosamide and Palbociclib reduce the viability of CCA cells, we treated CCLP cells with a Niclosamide concentration above the EC50 and a Palbociclib concentration below its EC50, resulting in a CI = 0.776 either individually or in combination for 72 h. We then assayed cell cycle progression and cell death using flow cytometry. Treatment of CCLP cells with Palbociclib and Niclosamide under these conditions resulted in a greater increase in the number of cells in G1 and a greater decrease in the number of cells in G2 than either treatment alone (Figure 5A). This is consistent with the decrease in phosphorylated RB shown in Figure 4C and Figure S8C,F. The combination of drugs also resulted in a decrease in the number of live cells and an increase in the number of apoptotic cells (Figure 5B). Very similar results were obtained using RBE cells and KKU-M055 cells (Figure S11). However, although KKU-M055 cells also showed a statistically significant increase in apoptosis with the combination treatment, RBE cells showed less pro-apoptotic effects. One reason for this could be that the RBE cells are less able to undergo apoptosis as they do not express detectable levels of cleaved caspase 3. We conclude that the combination of Niclosamide and Palbociclib results in increased cell cycle arrest in G2 and increased cell death, but the precise mechanism leading to increased cell death may not be the same in all cell types.

### 3.6. Combination Treatment Is More Effective than Single Agent Treatment In Vivo

**Figure 6:**
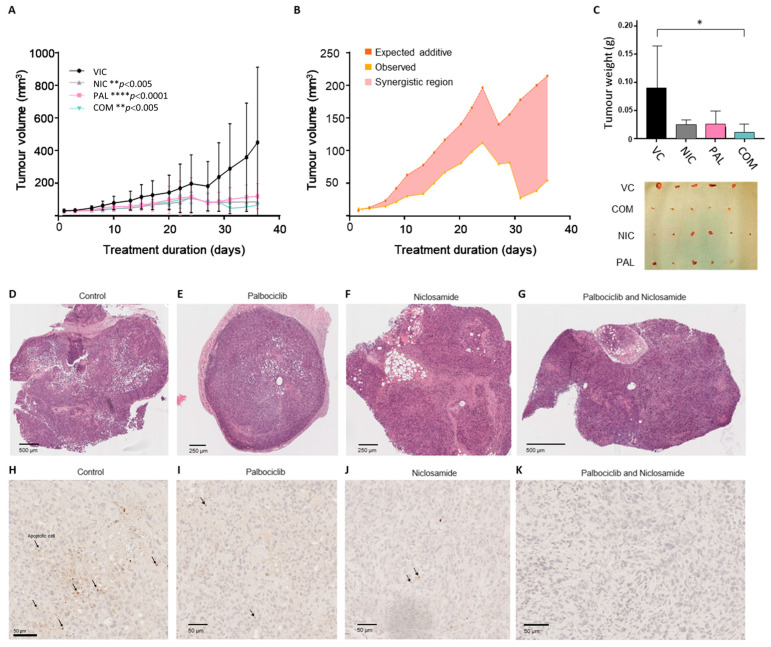
Niclosamide and Pablociclib act synergistically to reduce CCA tumour growth. (**A**) CCLP cells were injected subcutaneously into nude mice and allowed to grow until the tumours reached 3 mm in diameter. Four groups of mice were injected three times weekly intraperitoneally with vehicle control (VC), Niclosamide (NIC) (20 mg/kg), Palbociclib (PAL) (10 mg/kg), or both drugs (COM), and tumour volume was measured using callipers. **** *p* < 0.0001 ** *p* < 0.005 two-way ANOVA. (**B**) A comparison between the observed tumour volumes from the combination treatment and the expected additive tumour volumes over time. The observed combination tumour volume is the average tumour volume for the combination treatment group. The expected tumour volume (under the assumption of an additive effect) was calculated by using the Bliss Independence model: E_combination_ = V_PAL_ + V_NIC_ − [(V_PAL_ × V_NIC_)/V_control_], where V_PAL_, V_NIC_, and V_control_ are the average tumour volumes for Palbociclib, Niclosamide, and control groups, respectively. The shaded red region indicates synergistic effects, where the observed tumour volume is significantly lower than the expected additive effect. (**C**) Left—comparison of tumour weights across all treatment groups * *p* < 0.05 two-way ANOVA. Right—the excised tumours. (**D**–**G**) Representative images of haematoxylin and eosin-stained control and drug-treated xenograft sections (**H**–**K**). Representative images of control- and drug-treated xenograft sections stained for cleaved caspase 3 using IHC. The arrows highlight stained apoptotic cells.

To determine whether the combination of Niclosamide and Palbociclib is effective in a mouse model, we injected CCLP cells into immune deficient mice and allowed the tumours to grow until they reached 3 mm in diameter. The mice were then divided into 4 groups and treated with vehicle alone, Niclosamide alone, Palbociclib alone, or Palbociclib plus Niclosamide, for 30 days. At the dose used in this study, Niclosamide alone resulted in a decrease in tumour volume (Figure 6A). As expected, treatment with Palbociclib alone resulted in a decrease in tumour volume (Figure 6A). The combination treatment also dramatically decreased tumour volume (Figure 6A). Moreover, when the tumour volumes were analysed using the Bliss Independence model for drug synergy, the observed tumour volume for the combination of Niclosamide and Palbociclib was significantly lower than the expected tumour volume through additive effects, and thus the two drugs are considered synergistic (Figure 6B, Table S2). When the average tumour weights for the four groups of mice were compared, only the combination treatment resulted in a statistically significant difference from vehicle (Figure 6C). Importantly, observation of the excised tumours showed that 5/5 control vehicle-treated tumours were >150 mm^3^, whereas only 2/5 single-agent-treated tumours were >150 mm^3^, and 0/5 combination-treated tumours were >150 mm^3^. Histological analysis demonstrated viable tumour cells within the xenografts across most of the samples (Figure 6D–G). Tumours were well-circumscribed from host tissue and showed variable amounts of necrosis. Occasional bands of fibrosis were present, and in places, islands of sclerosis had formed within and adjacent to the tumour. No relationship between treatment condition and levels of necrosis/fibrosis was observed. To evaluate whether treatment with Palbociclib or Niclosamide or both led to increased apoptosis at the termination of the experiment, immunohistochemistry was performed using an anti-cleaved caspase-3 antibody (Figure 6F–K). Apoptotic cells (demonstrated by immunoreactivity with this antibody) was systematically evaluated across the samples. However, no statistically significant relationship between treatment with these compounds and apoptosis was demonstrated (Figure S12). We conclude that, in this model, the combination of Niclosamide and Palbociclib provides an added benefit over single-agent treatment. However, this is not accompanied by an observable increase in apoptosis or necrosis in the tumours, at least after 30 days of treatment.

## 4. Discussion

Drug repurposing is a pragmatic approach for treatment as it can allow a more rapid clinical impact at a lower cost than de novo drug discovery and development [34]. A drug repurposing strategy to screen a library of off-patent drugs/agents with low toxicity, at clinically relevant peak serum concentrations, was used to identify drugs for treatment of multiple myeloma [20]. Here we screened the same library using CCA cells and identified Niclosamide as a potent inhibitor of CCA cell viability. Dose–response studies using CCA cell lines with diverse aetiology and mutational backgrounds were used in this study (RBE, KKU-M055, and CCLP) and showed EC50 values between 0.39 and 1.31 μM. Importantly, while Niclosamide also reduced viability of normal primary biliary epithelial cells (BECs), these cells were less sensitive compared to CCA cells. Niclosamide is known to depolarise the mitochondrial membrane by moving protons to the mitochondrial matrix and decoupling the electron transport chain, thus blocking ATP synthesis and initiating cytochrome C dependent apoptosis; in addition, it inhibits multiple signalling pathways [21]. CCA cell lines that are derived from liver-fluke–infected patients have been shown to be sensitive to Niclosamide through mitochondrial membrane depolarisation, as a consequence of alterations in metabolism leading to an elevation of niacinamide, suppression of NAD^+^/NADH ratio, and ATP depletion [24]. This study reveals that Niclosamide sensitivity is not confined to liver-fluke–derived CCA and that Niclosamide is an inhibitor of CCA cells with very different mutational profiles.

Niclosamide treatment decreased the expression of PRH in CCA cells, and this protein has previously been shown to act as an oncoprotein in these cells [16]. Treatment with the proteasome inhibitor MG132 abrogated Niclosamide-induced PRH degradation, suggesting proteasomal processing or degradation. However, PRH knockout in CCLP cells showed that the effect of Niclosamide on cell viability does not require PRH. This demonstrates that, in these cells, loss of PRH is not the only action of Niclosamide that decreases cell viability. Moreover, Niclosamide treatment also decreased β-catenin and Cyclin D1 protein levels in CCA cell lines and increased global protein ubiquitinylation as well as bringing about mitochondrial uncoupling. Further experiments will be needed to determine which proteins and pathways are essential to mediate the effects of Niclosamide on these cells.

Niclosamide has been suggested as a potential treatment for many types of cancer, particularly p53 defective cancers [21,35,36]. However, Niclosamide has poor water solubility, a relatively low absorption rate, and low bioavailability, and it can induce non-specific cytotoxicity (reviewed [35]). In CCA cells, a longer incubation with the drug was able to achieve selective inhibition of cancers cells over BECs. This imposes limitations to using Niclosamide in the clinic. To improve the amount of drug that reaches the tumour, Niclosamide chemistry and administration have been investigated. Niclosamide derivatives have been optimised to improve aqueous solubility whilst maintaining the anti-cancer activity of Niclosamide [37]. Nano-suspension of Niclosamide was shown to improve the oral bioavailability of the drug [38]. Niclosamide has been used in early clinical trials against castration-resistant prostate cancer in combination with abiraterone and prednisone, where the solubility and absorption of Niclosamide was increased by suppling the powdered drug in capsules [39]. The serum concentration was kept constant between administrations and was maintained in the 0.31 μM-0.65 μM range. Also, to reduce the amount of Niclosamide needed to produce an effect, Niclosamide has been studied in combination with multiple chemotherapy regimens, targeted therapy, as well as radiotherapy to induce additive or synergistic effects [22].

Combination therapy is thought to be an easier way of transferring Niclosamide treatment to the clinic for multiple cancer types. We tested the combination of Niclosamide with the CDK4/6 inhibitor Palbociclib in the expectation that these inhibitors might act synergistically. Palbociclib is a first-line treatment for Hormone Receptor–positive HER2- breast cancer [40], and it is in clinical trials for a variety of other cancers, including non-small-cell lung cancer [41]. Therefore, any drugs, which can potentiate the actions of Palbociclib, are of clinical interest. The rationale for investigating this combination is that both drugs were able to exert selective targeting of CCA cell lines over BECs, thus the combination of both drugs may lead to a more potent anti-CCA formulation. Also, as both Niclosamide and Palbociclib have well-documented drug profiles in humans, the aims of pursuing this combination in patients is realistic and translatable. Palbociclib has been previously investigated in a small number of CCA patients, but had no obvious benefit [42]. However, we reasoned that its activity could be improved by dual therapy. For the combination treatment, the Chou–Talalay method resulted in combination index values below 1 in CCLP, RBE, and KKU-M055 CCA cells, indicating drug synergism. In support of this, Western blotting showed that both drugs reduced phosphorylated RB protein (pRB) levels, and their combination had a stronger effect. Palbociclib is well known to suppress RB phosphorylation; however, for Niclosamide, this is a novel finding. As discussed previously, this could be related to a Niclosamide-induced ubiquitin-mediated proteasomal degradation mechanism. Wong et al. report a synergism between Palbociclib and PI3K inhibitors [43]. They hypothesised that as Palbociclib resistance is associated with Cyclin D up-regulation, they could counteract this increase in expression with PI3K/mTOR inhibition. They showed that a Palbociclib-induced increase in Cyclin D3 was abolished by Omipalisib (PI3K/mTOR inhibitor) [43]. In this work, we report similar findings with Cyclin D1 expression in CCA cell lines in that Niclosamide treatment decreased Cyclin D1 expression and acts synergistically with Palbociclib. To examine the mechanistic basis of this synergy, alternative drugs within the same classes were tested. Substitution of Palbociclib with the CDK4/6 inhibitor Ribociclib and substitution of Niclosamide with the mitochondrial uncoupler FCCP also resulted in synergistic effects, suggesting that cell cycle inhibition combined with mitochondrial depolarization is responsible for synergy in this case. The Cyclin B1/CDK1 complex links cell cycle control and mitochondrial membrane depolarisation. In fact, Cyclin B/CDK1 phosphorylates complex I of the electron transport chain and activates it at the G2/M transition to enable increased energy production for successful cell-cycle progression through mitosis [44]. The mitochondrial membrane is transiently depolarised at telophase [45]. Thus, perturbation of cell cycle by Palbociclib and downstream effects on CDK1 activity could increase the effects of Niclosamide, arrest cell division, and promote cell death. Further investigation is required to determine the precise molecular basis of synergy.

To assess whether the combination of Niclosamide and Palbociclib could be efficacious in models that more closely resemble tumours in patents, we made use of CCLP and BEC spheroids as well as a mouse xenograft tumour model. The combination of Niclosamide and Palbociclb reduced the size of CCLP spheroids and reduced the viability of CCLP cells grown under these conditions. However, single treatments and the combination treatment had little or no effect on the size or viability of BEC spheroids. Moreover, in a xenograft mouse model, while both drugs reduced tumour growth, the combination treatment resulted in a greater reduction in tumour weight, and Bliss Independence modelling confirmed that there was a synergistic effect. We did not observe increased apoptosis or increased necrosis in the treated tumours, and although this may be due to the fact that the tumours were examined after 30 days of treatment, further experiments will be required to determine the precise molecular basis for synergism.

## 5. Conclusions

This work shows that Niclosamide is a potent inhibitor of CCA cell viability and that this drug acts through multiple mechanisms. Moreover, we demonstrate the ability of Niclosamide to act synergistically with CDK4/6 inhibition. These preclinical studies support further investigation into the use of Niclosamide alone and Niclosamide combined with Palbociclib as a potential therapy for CCA.

## Figures and Tables

**Table 1:** Spacer sequences of sgRNAs targeting *HHEX* used in this study.

Guide Name	Spacer Sequencer (5′–3′)	PAM	Strand	On-Target Score	Off-Target Score
Exon 1 Left	AGAGCUGCUGGCCCGCGCCG	CGG	−	48.4	42.9
Exon 1 Right	GCCAUGCAGUACCCGCACCC	CGG	+	54.6	42.6
Exon 2 Left	GAAUUUCUUCUCCAGCUCGA	TGG	−	52.5	40.2
Exon 2 Right	AGAGACAGGUGAGCUCGCGG	GGG	+	61.8	45.5

**Table 2:** Sequences of oligonucleotides annealed and ligated into pX461 to encode for sgRNAs targeting HHEX.

Target Site		Sequence (5′–3′)
Exon 1 Left	Top	CACCGAGAGCTGCTGGCCCGCGCCG
	Bottom	AAACCGGCGCGGGCCAGCAGCTCT
Exon 1 Right	Top	CACCGGCCATGCAGTACCCGCACCC
	Bottom	AAACGGGTGCGGGTACTGCATGGC
Exon 2 Left	Top	CACCGGAATTTCTTCTCCAGCTCGA
	Bottom	AAACTCGAGCTGGAGAAGAAATTC
Exon 2 Right	Top	CACCGAGAGACAGGTGAGCTCGCGG
	Bottom	AAACCCGCGAGCTCACCTGTCTCT

**Table 3:** Relative EC50 values for CCA cell lines (CCLP, RBE, and KKU-M055) and primary biliary epithelial cells (BECs) treated with Niclosamide.

EC50 (mM)	BECs	CCLP	RBE	KKU-M055
72 h	2.55	0.45	1.31	0.39
24 h	0.82	1.33	1.11	0.75

## Data Availability

All data included in this study are available upon request from the corresponding authors.

## Supplementary Materials

The following supporting information can be downloaded at: https://www.mdpi.com/article/10.3390/cancers17223721/s1, Figure S1: Drug screening and drug validation. Figure S2: Quantification of PRH, β-catenin, Cyclin D1, and cleaved caspase 3 protein levels in CCLP cells after treatment with Niclosamide. Figure S3: Niclosamide treatment decreases the levels of PRH and Cyclin D1 in RBE and KKU-M055 cells and increase the levels of cleaved caspase3 in KKU-M055 cells. Figure S4: Quantification of global protein ubiquitination in CCA cell lines. Figure S5: Proteasome inhibition restores PRH protein levels in RBE cells. Figure S6: PRH knockout does not reduce sensitivity to Niclosamide. Figure S7: Palbociclib treatment for 24 h decreases the viability of CCA cells. Figure S8: Niclosamide and Pablociclib act synergistically to reduce the viability or RBE and KKU-M055 CCA cells. Figure S9: Niclosamide and Pablociclib reduce the levels of phosphorylated RB and PRH in CCA cell lines. Figure S10: Ribocilib and FCCP can replace Palbociclib and Niclosamide, respectively, to synergistically reduce CCLP cell viability. Figure S11: Combining Niclosamide and Pablociclib results in decreased cells in G2 and increased apoptosis in RBE cells and KKU-055 cells. Figure S12: Analysis of apoptotic cells in xenograft tumour samples. Table S1: Drug list. Table S2: Observed tumour volumes from the combination treatment and the expected additive tumour volumes over time. File S1: Original western blots.
