# Supplementary material for: Niclosamide and Palbociclib Act Synergistically to Reduce Cholangiocarcinoma Cell Viability In Vitro and Inhibit Tumour Growth in a Mouse Model
Source: Cancers (Basel). 2025 Nov 20;17(22):3721. doi: 10.3390/cancers17223721 (PMC12651616; doi:10.3390/cancers17223721)
Supplement: Supplementary file 1 [file cancers-17-03721-s001.zip › File S1.pptx]

## Slide 1
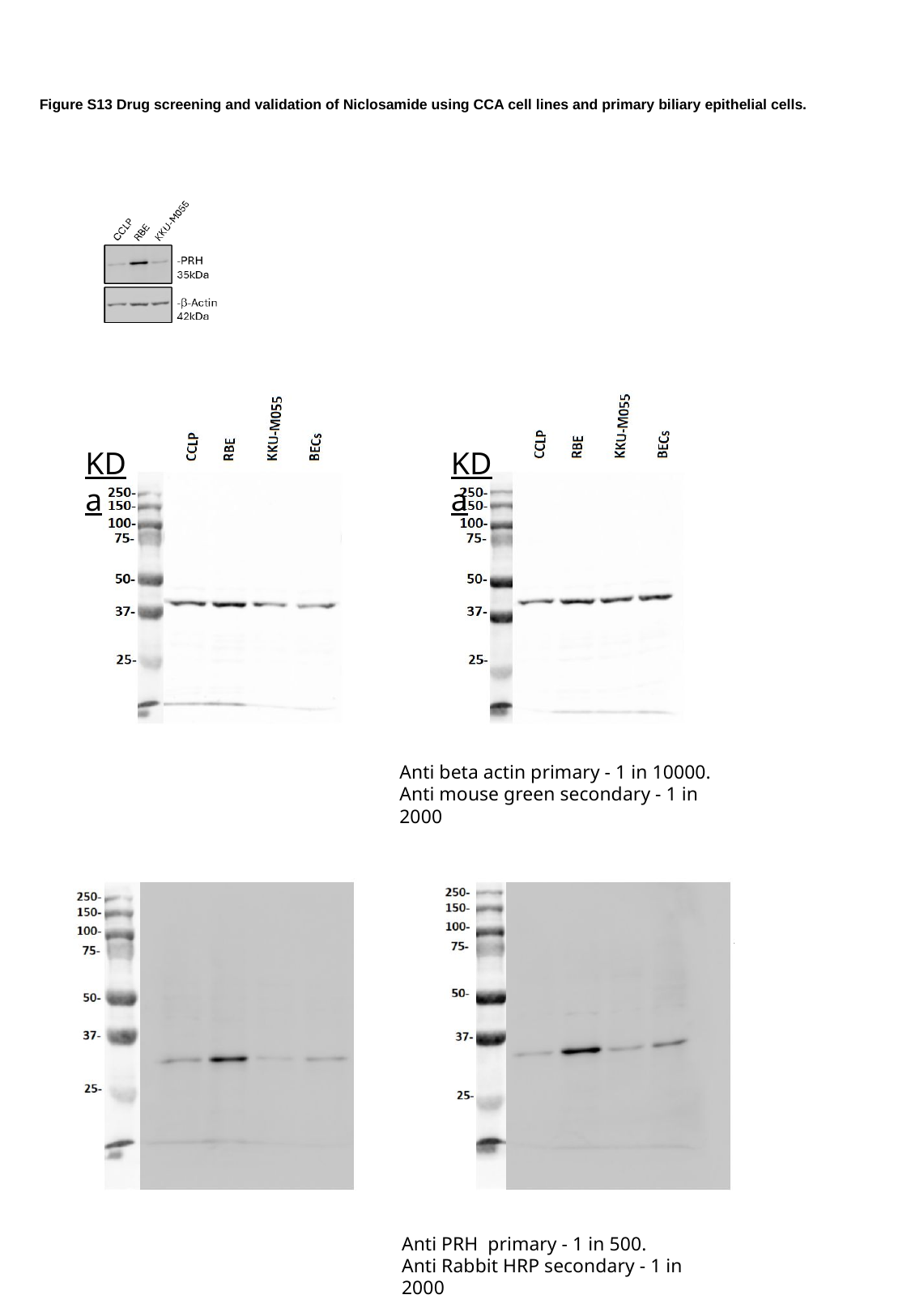

Figure S13 Drug screening and validation of Niclosamide using CCA cell lines and primary biliary epithelial cells.
KDa
KDa
Anti beta actin primary - 1 in 10000.
Anti mouse green secondary - 1 in 2000
Anti PRH primary - 1 in 500.
Anti Rabbit HRP secondary - 1 in 2000

## Slide 2
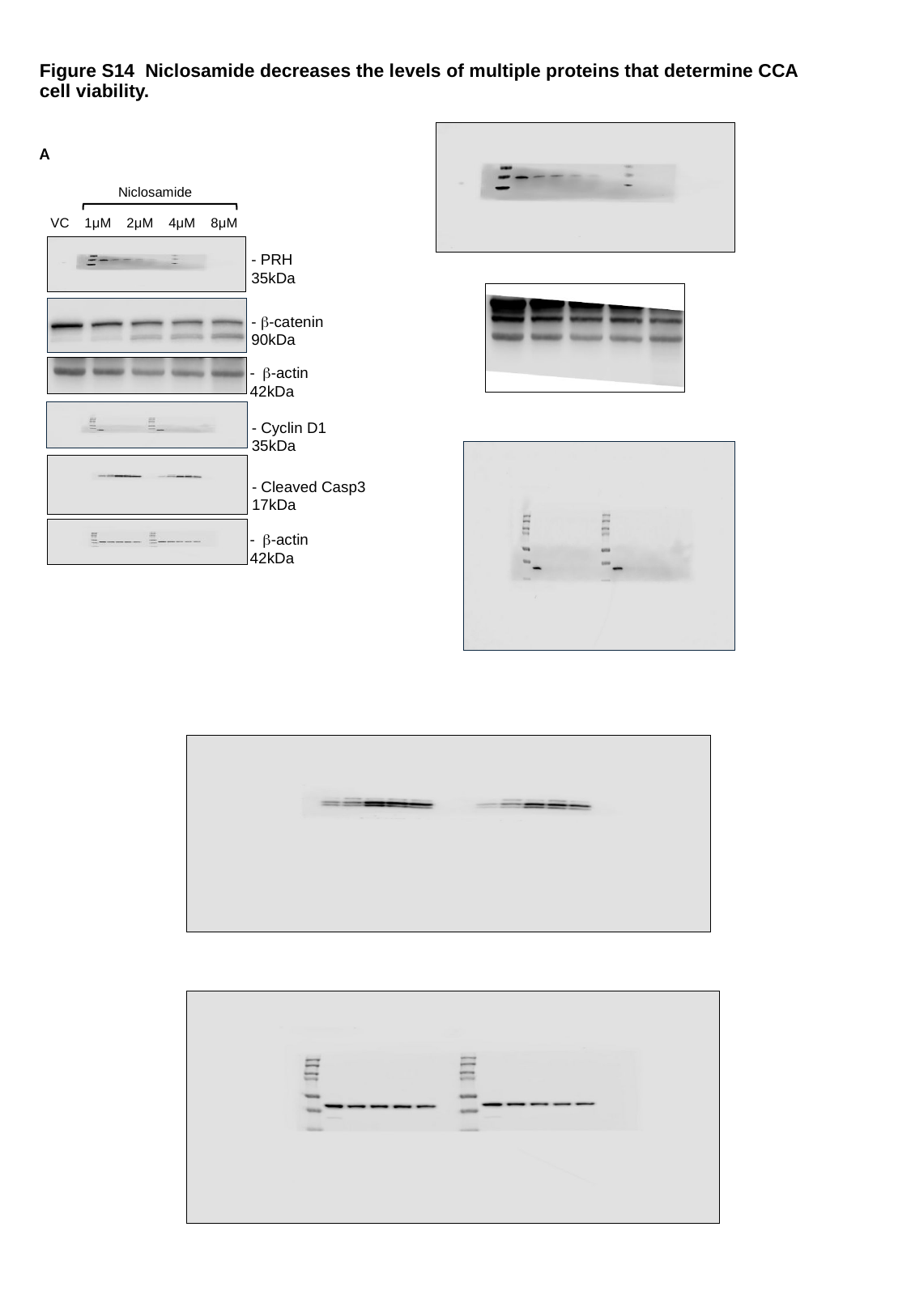

Figure S14 Niclosamide decreases the levels of multiple proteins that determine CCA cell viability.
A
Niclosamide
VC 1μM 2μM 4μM 8μM
- PRH
35kDa
- b-catenin
90kDa
- b-actin
42kDa
- Cyclin D1
35kDa
- Cleaved Casp3
17kDa
- b-actin
42kDa

## Slide 3
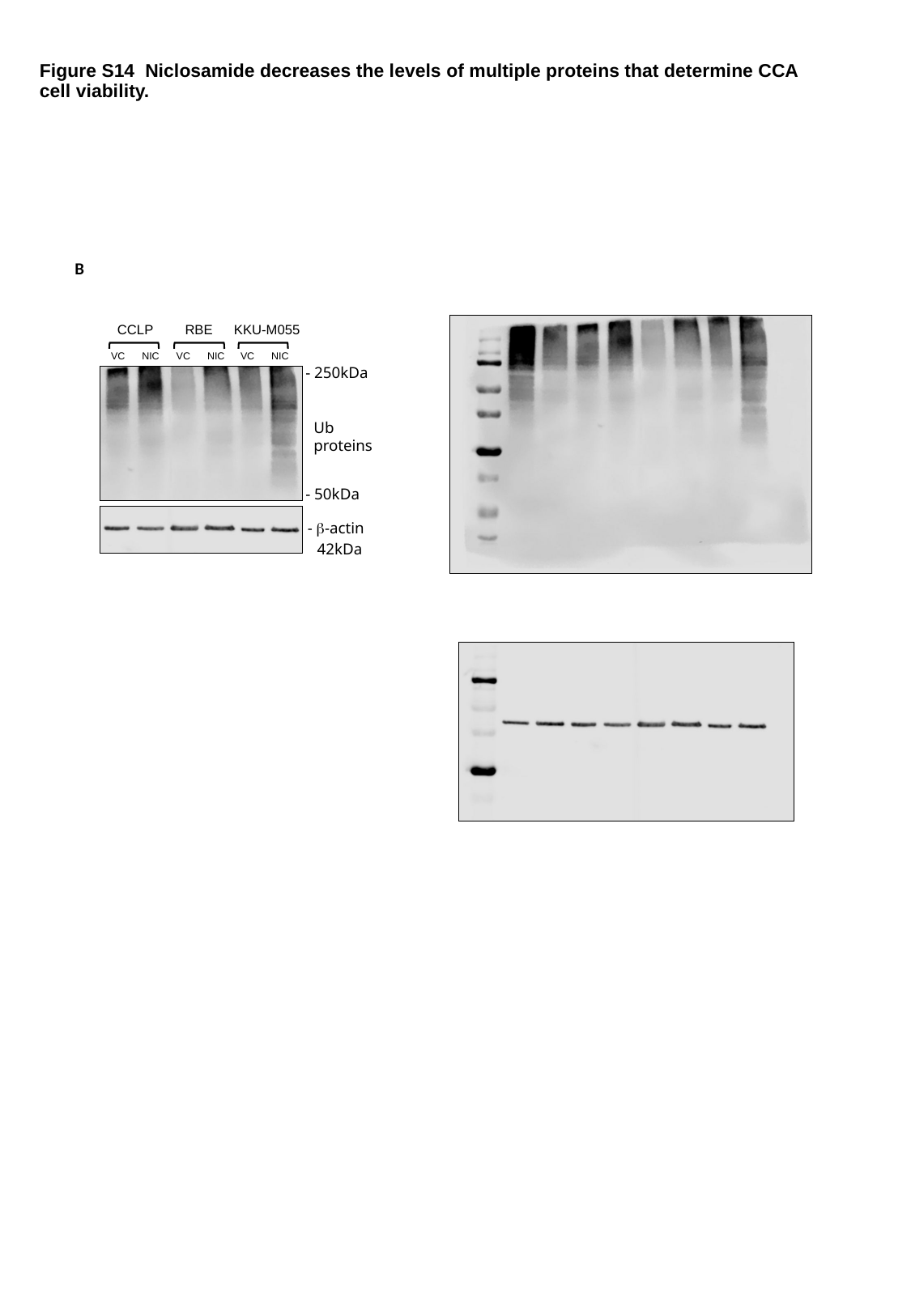

Figure S14 Niclosamide decreases the levels of multiple proteins that determine CCA cell viability.
B
CCLP
RBE
KKU-M055
VC NIC
 VC NIC
VC NIC
- 250kDa
 Ub
 proteins
- 50kDa
- b-actin
 42kDa

## Slide 4
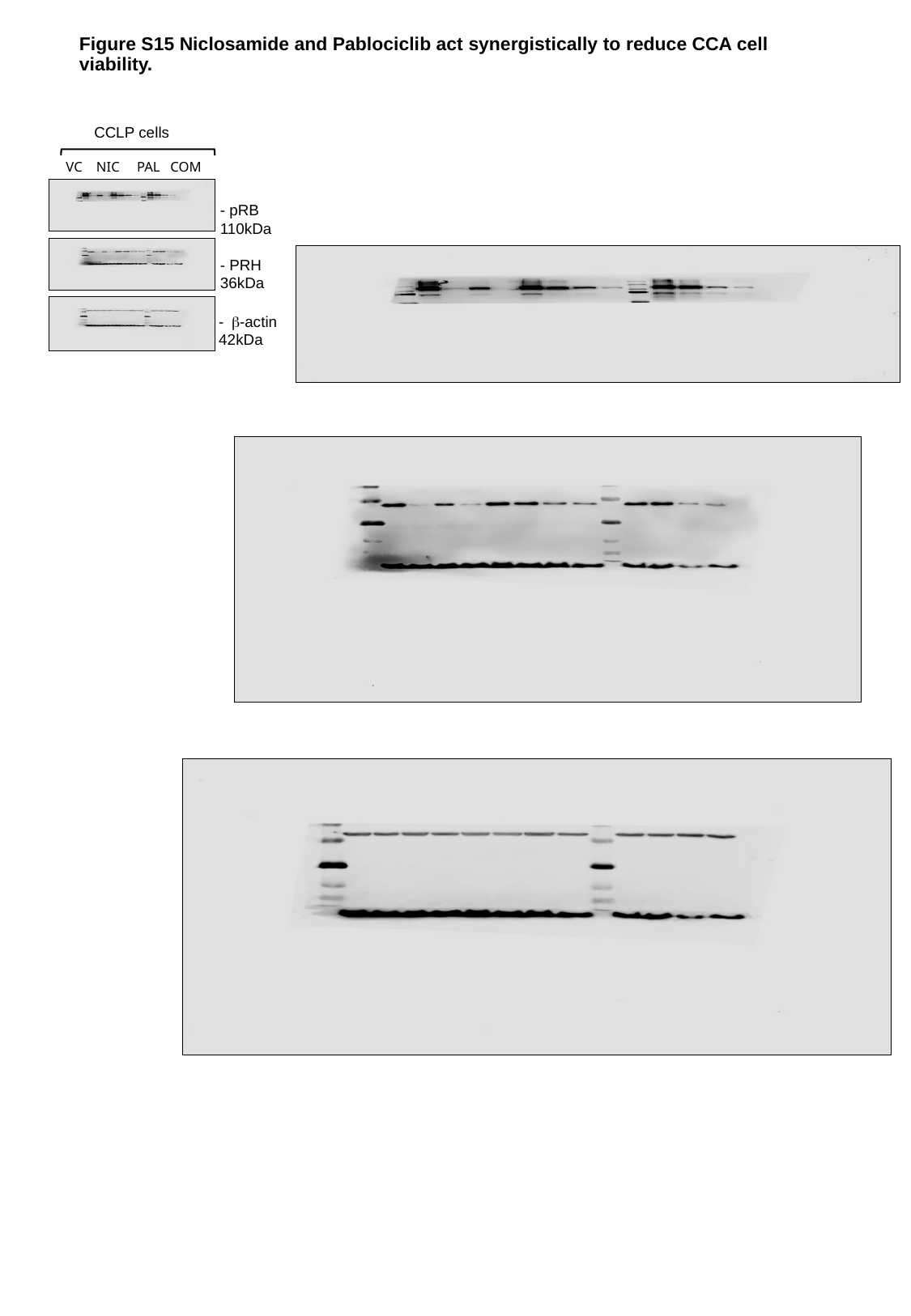

# Figure S15 Niclosamide and Pablociclib act synergistically to reduce CCA cell viability.
CCLP cells
 VC NIC PAL COM
- pRB
110kDa
- PRH
36kDa
- b-actin
42kDa

## Slide 5
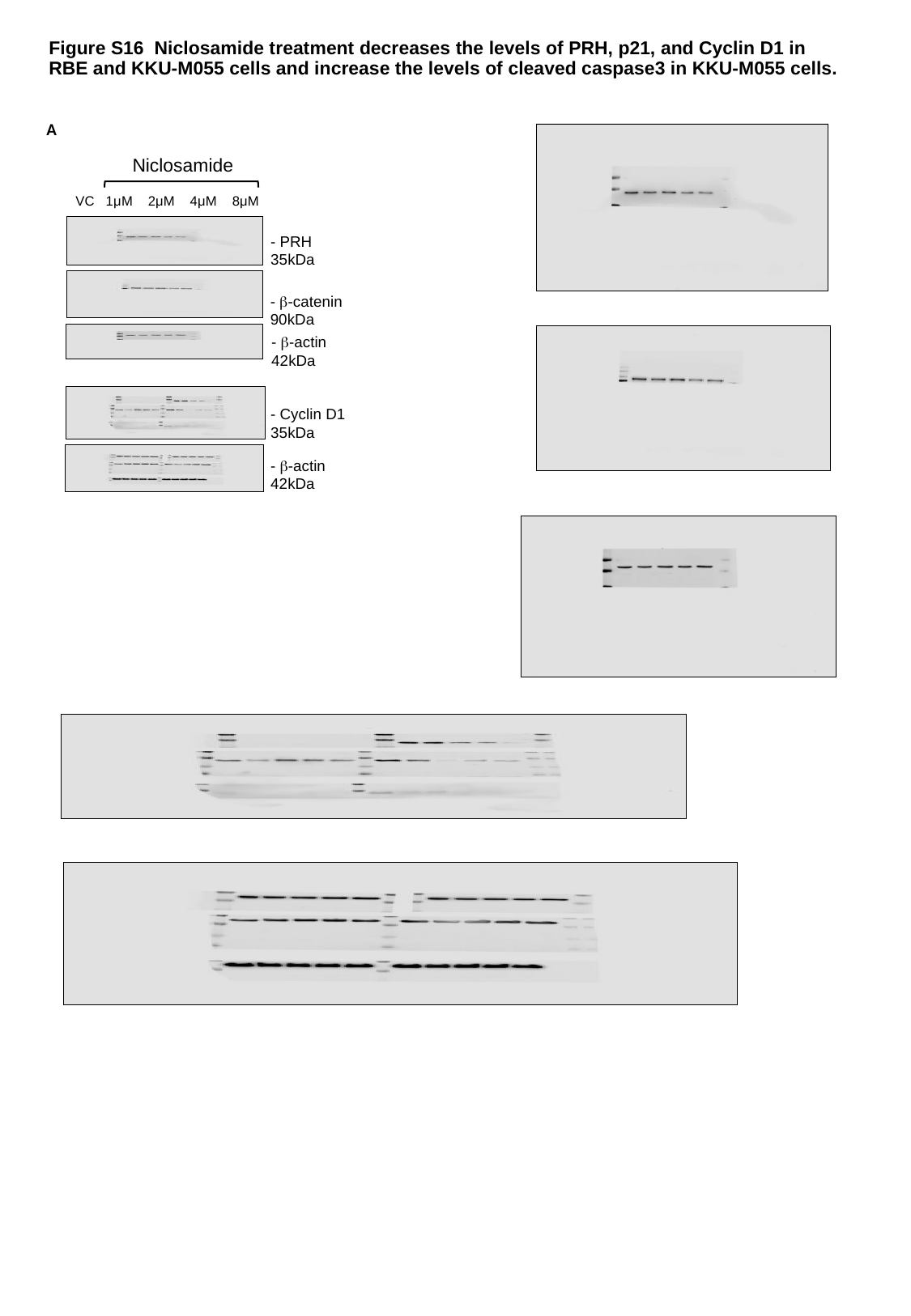

Figure S16 Niclosamide treatment decreases the levels of PRH, p21, and Cyclin D1 in RBE and KKU-M055 cells and increase the levels of cleaved caspase3 in KKU-M055 cells.
A
Niclosamide
VC 1μM 2μM 4μM 8μM
- PRH
35kDa
- b-catenin
90kDa
- b-actin
42kDa
- Cyclin D1
35kDa
- b-actin
42kDa

## Slide 6
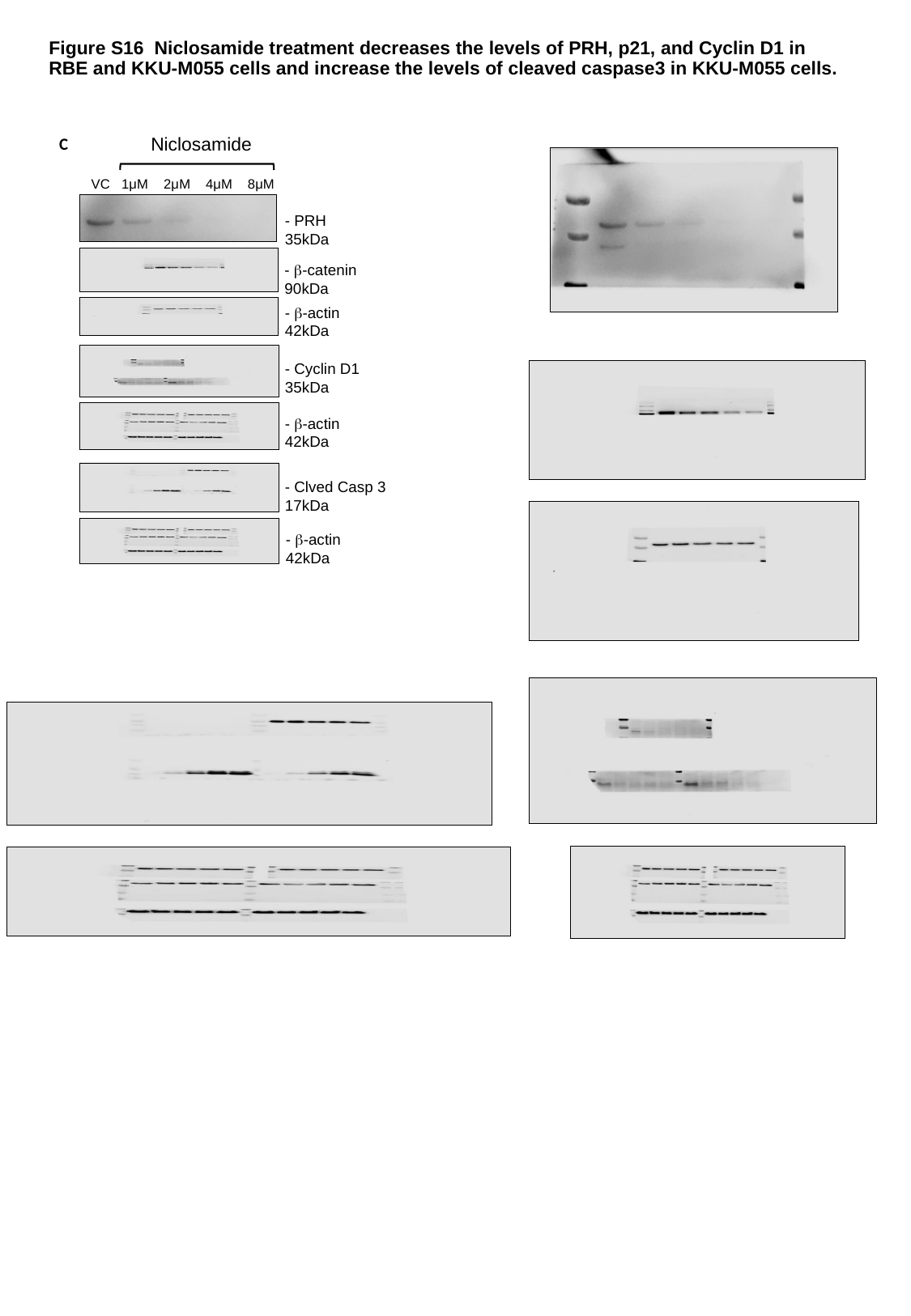

Figure S16 Niclosamide treatment decreases the levels of PRH, p21, and Cyclin D1 in RBE and KKU-M055 cells and increase the levels of cleaved caspase3 in KKU-M055 cells.
Niclosamide
C
VC 1μM 2μM 4μM 8μM
- PRH
35kDa
- b-catenin
90kDa
- b-actin
42kDa
- Cyclin D1
35kDa
- b-actin
42kDa
- Clved Casp 3
17kDa
- b-actin
42kDa

## Slide 7
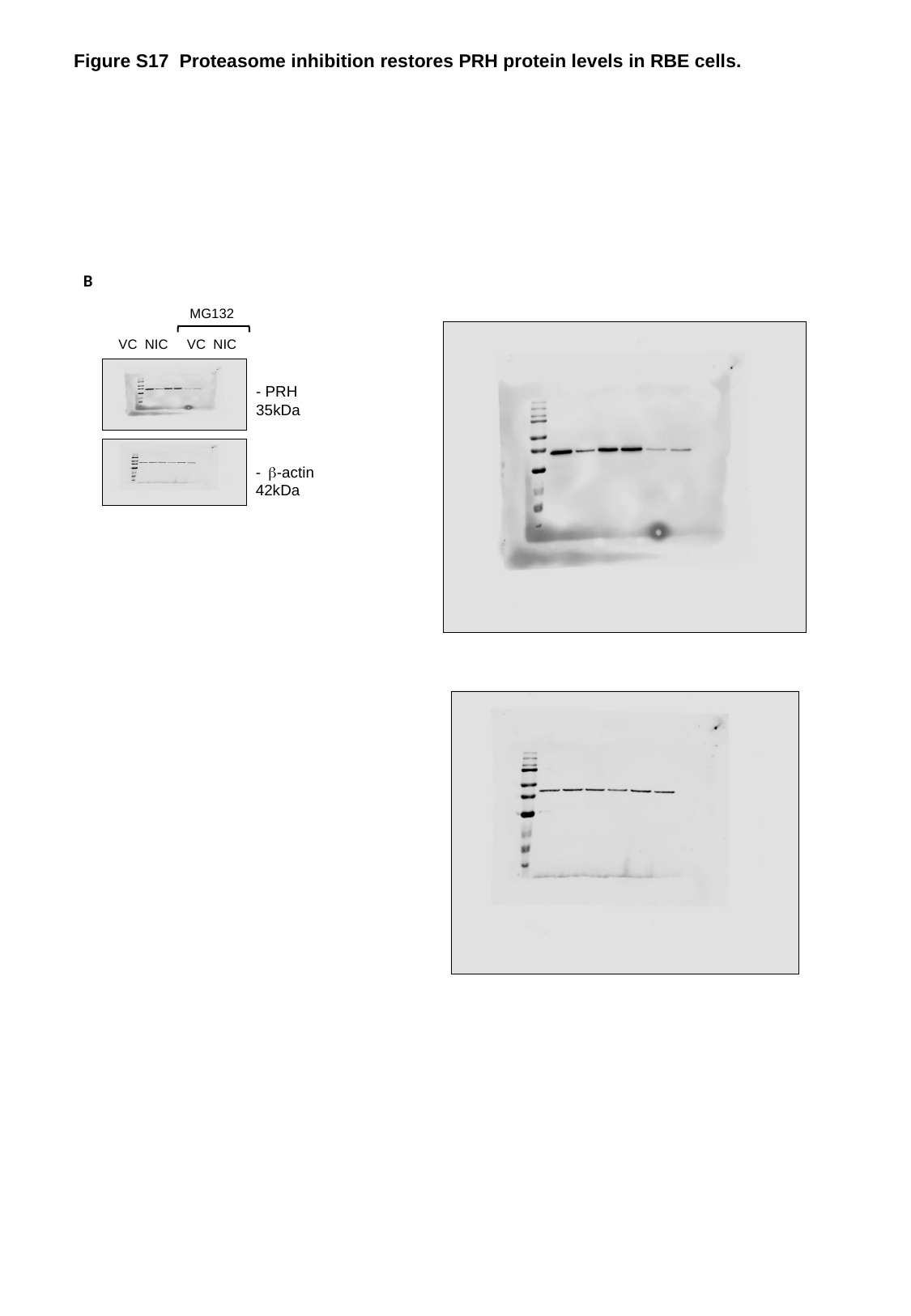

Figure S17 Proteasome inhibition restores PRH protein levels in RBE cells.
B
MG132
VC NIC
VC NIC
- PRH
35kDa
- b-actin
42kDa

## Slide 8
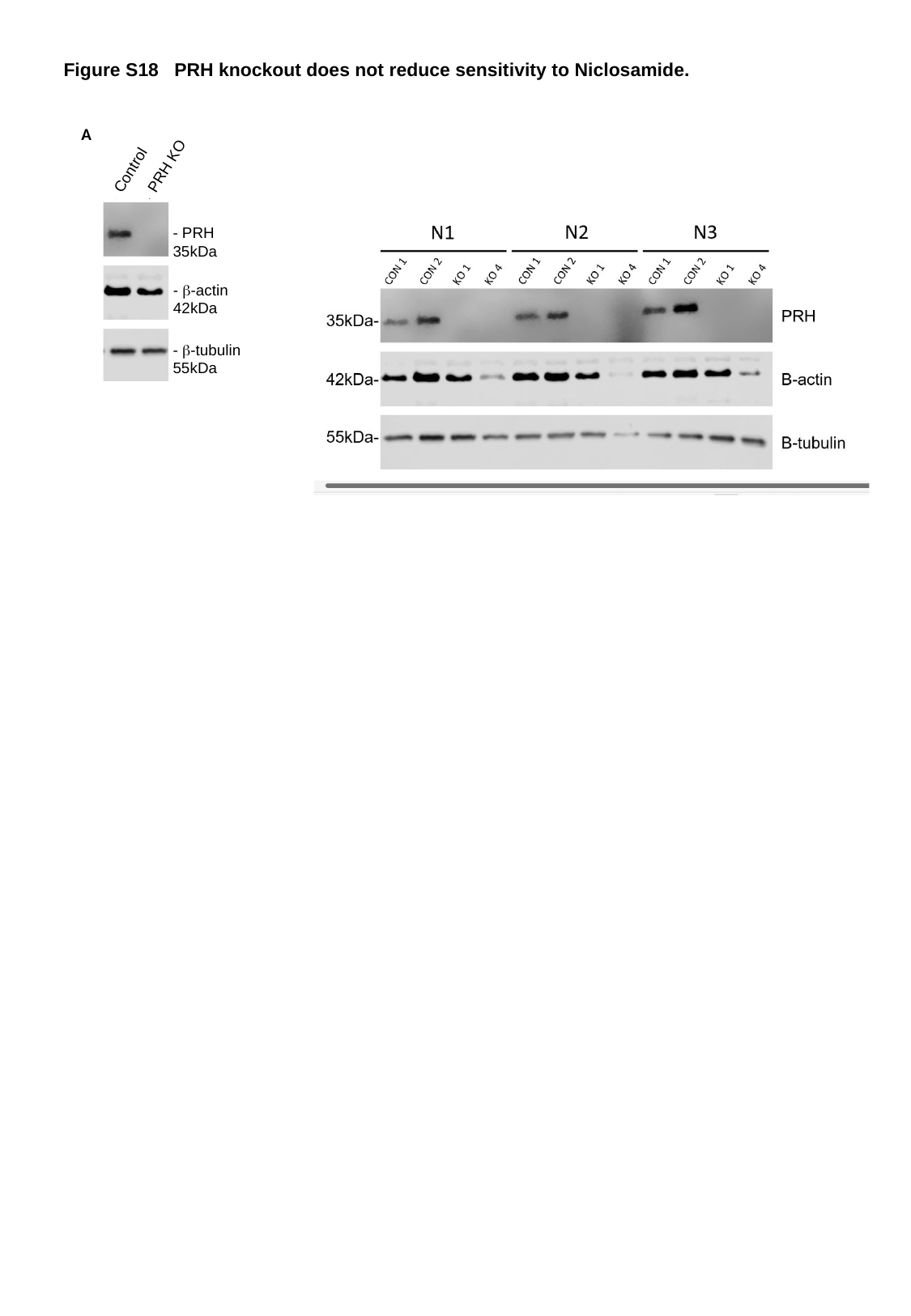

Figure S18 PRH knockout does not reduce sensitivity to Niclosamide.
Control
PRH KO
- PRH
35kDa
- b-actin
42kDa
- b-tubulin
55kDa
A

## Slide 9
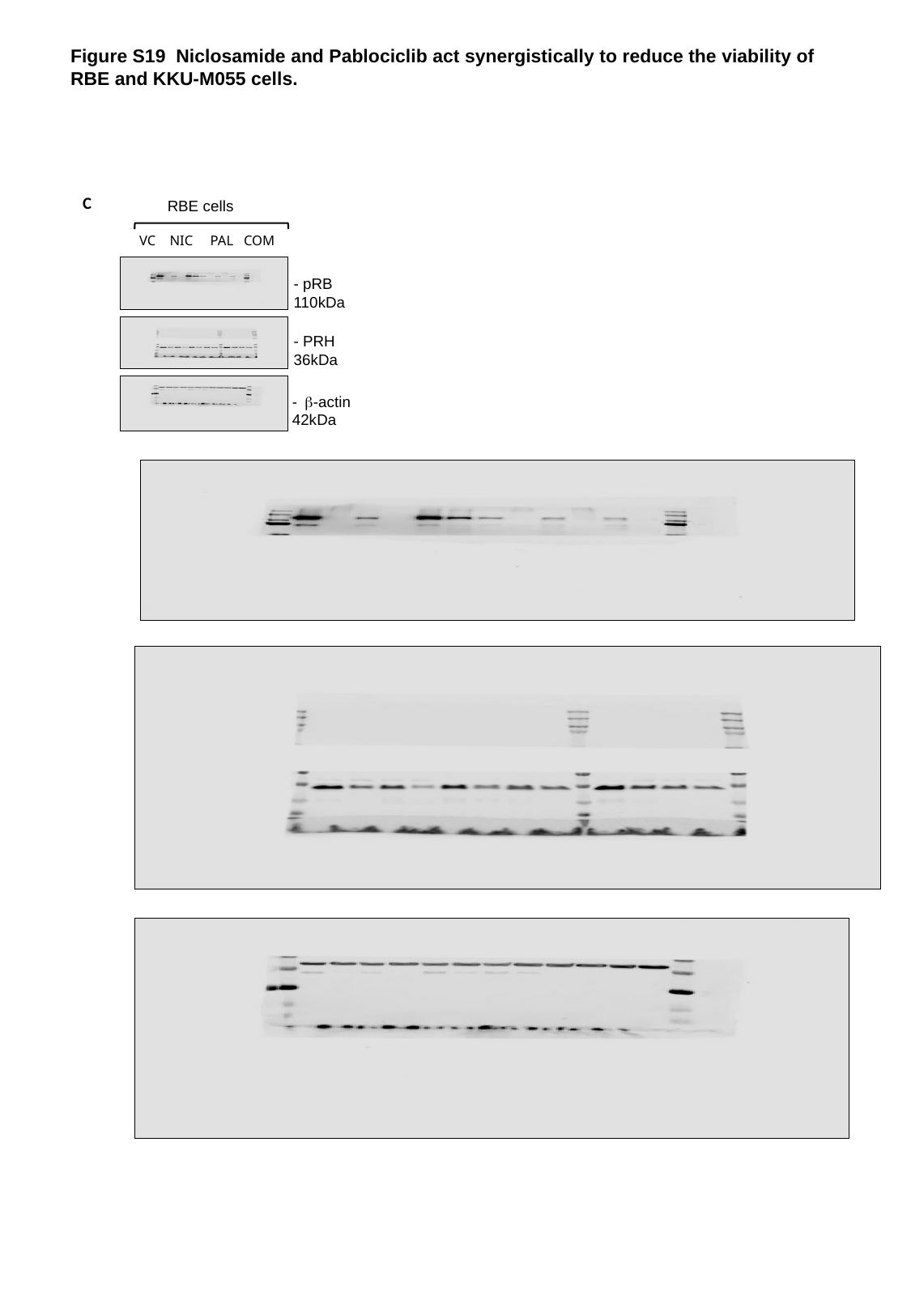

# Figure S19 Niclosamide and Pablociclib act synergistically to reduce the viability of RBE and KKU-M055 cells.
C
RBE cells
 VC NIC PAL COM
- pRB
110kDa
- PRH
36kDa
- b-actin
42kDa

## Slide 10
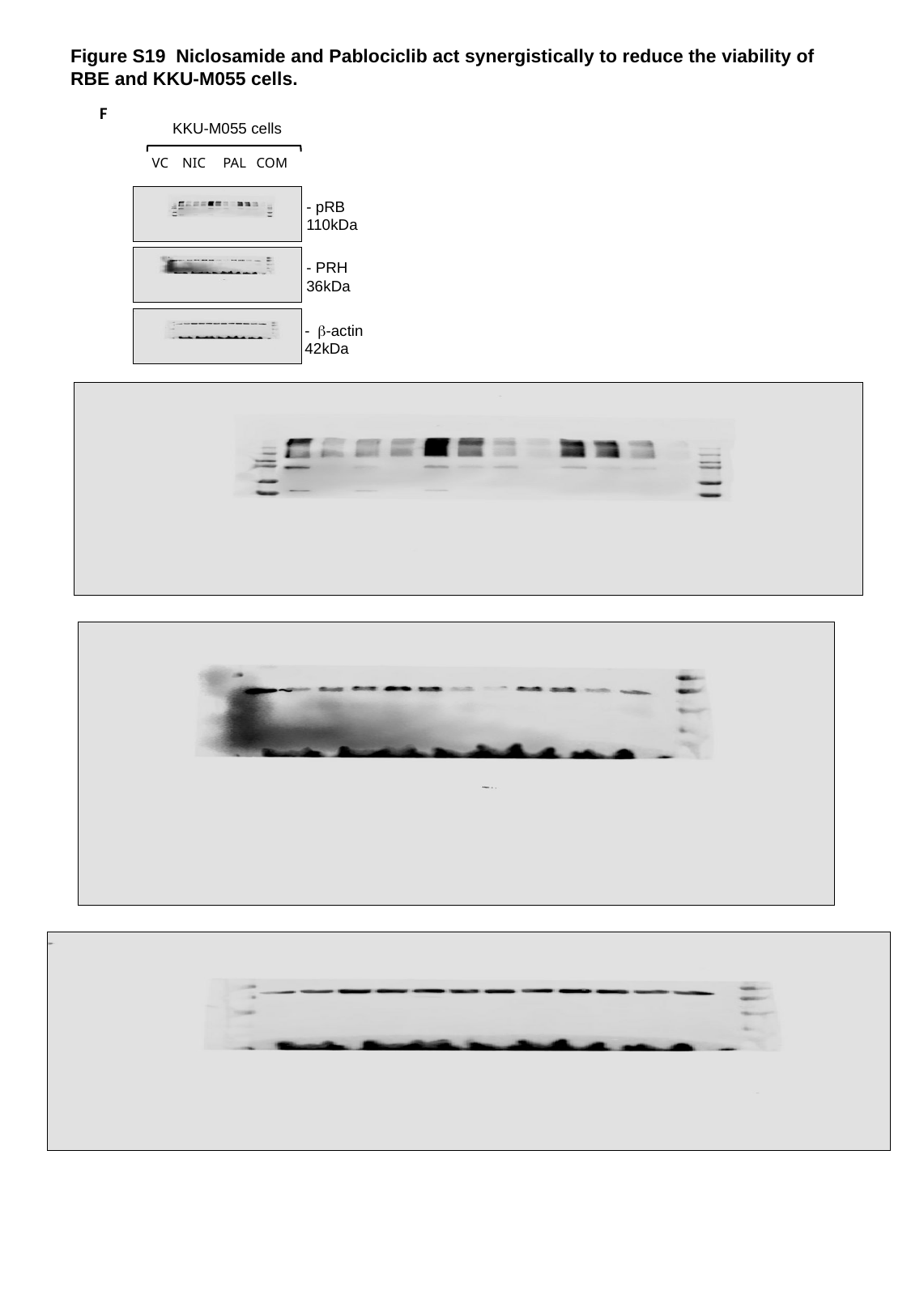

# Figure S19 Niclosamide and Pablociclib act synergistically to reduce the viability of RBE and KKU-M055 cells.
F
KKU-M055 cells
 VC NIC PAL COM
- pRB
110kDa
- PRH
36kDa
- b-actin
42kDa
