# Supplementary material for: Niclosamide and Palbociclib Act Synergistically to Reduce Cholangiocarcinoma Cell Viability In Vitro and Inhibit Tumour Growth in a Mouse Model
Source: Cancers (Basel). 2025 Nov 20;17(22):3721. doi: 10.3390/cancers17223721 (PMC12651616; doi:10.3390/cancers17223721)
Supplement: Supplementary file 1 [file cancers-17-03721-s001.zip › Supplementary material.pptx]

## Slide 1
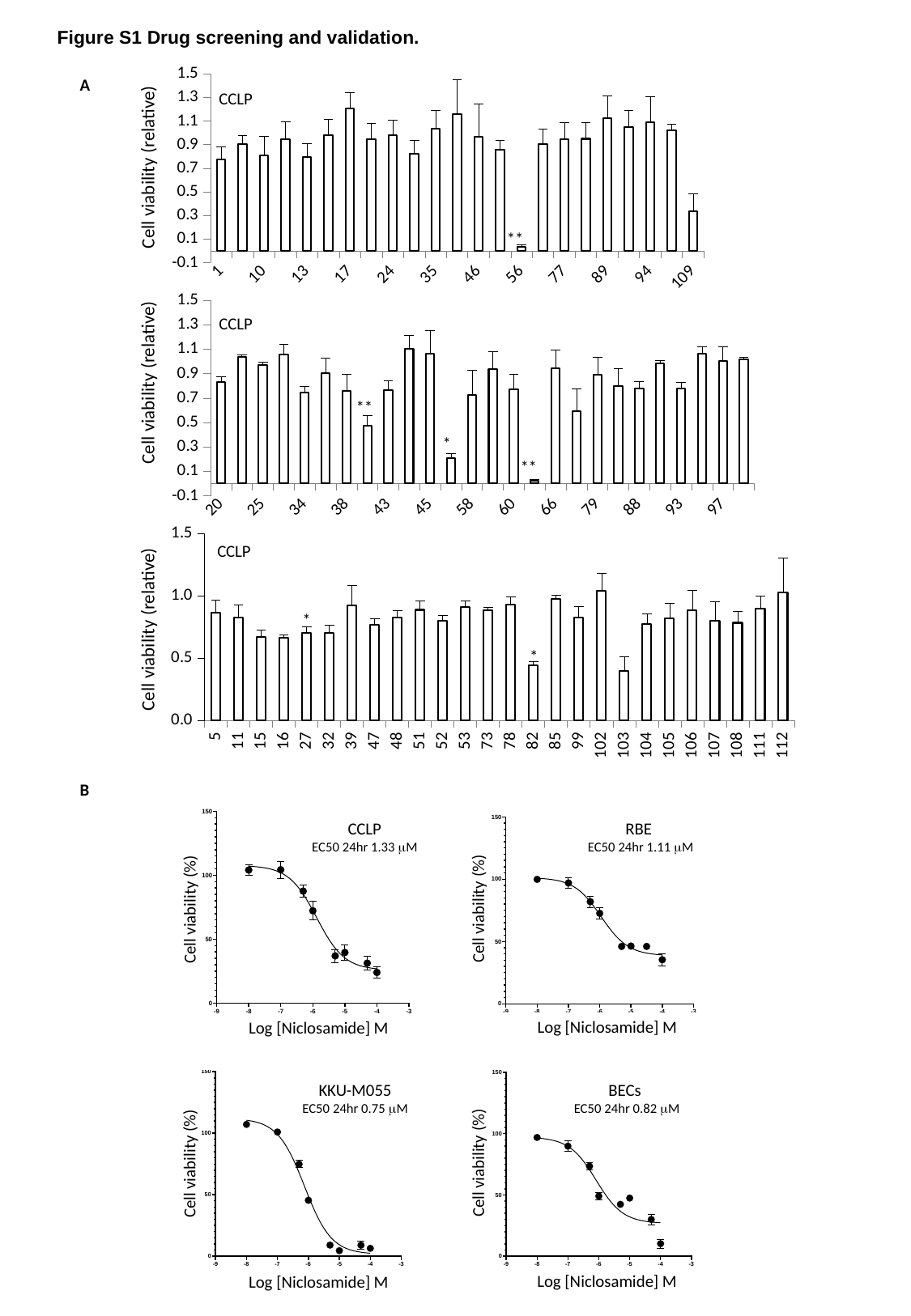

# Figure S1 Drug screening and validation.
### Chart
| Category | |
|---|---|
| 1 | 0.7760076609203219 |
| 7 | 0.902199306903592 |
| 10 | 0.8101744644432848 |
| 12 | 0.9474117454452946 |
| 13 | 0.7925657992894535 |
| 14 | 0.9825228601055489 |
| 17 | 1.2050585524311703 |
| 22 | 0.9487119417467241 |
| 24 | 0.9790212974068214 |
| 33 | 0.8217674516404422 |
| 35 | 1.0370416183319346 |
| 40 | 1.1582970802247832 |
| 46 | 0.9664411634123232 |
| 50 | 0.8581354128762523 |
| 56 | 0.035448273786883644 |
| 76 | 0.9061048155061154 |
| 77 | 0.9469567905148151 |
| 87 | 0.9492633125544999 |
| 89 | 1.125150222061684 |
| 92 | 1.047790700255491 |
| 94 | 1.0899736491839904 |
| 98 | 1.0186831632995474 |
| 109 | 0.33454779133750767 |A
CCLP
Cell viability (relative)
### Chart
| Category | |
|---|---|
| 5 | 0.8647044544406247 |
| 11 | 0.8222625115815481 |
| 15 | 0.6678997677202719 |
| 16 | 0.6636955648733278 |
| 27 | 0.7034385181673359 |
| 32 | 0.7027855472349489 |
| 39 | 0.9200550205038244 |
| 47 | 0.7633908376046964 |
| 48 | 0.8247243593426581 |
| 51 | 0.8857902004829575 |
| 52 | 0.7987912813932073 |
| 53 | 0.9083653849631147 |
| 73 | 0.8848866943741083 |
| 78 | 0.9291968838899706 |
| 82 | 0.43978928388870125 |
| 85 | 0.9747712023955928 |
| 99 | 0.8265380160714143 |
| 102 | 1.0358846299223055 |
| 103 | 0.3962623122005631 |
| 104 | 0.7726314718955395 |
| 105 | 0.818972898159208 |
| 106 | 0.8847781138805999 |
| 107 | 0.7952571505509343 |
| 108 | 0.781951605530692 |
| 111 | 0.8967316042728134 |
| 112 | 1.026773201631663 |*
*
**
### Chart
| Category | |
|---|---|
| 20 | 0.8330458191243076 |
| 21 | 1.0390474982077071 |
| 25 | 0.9681800473554293 |
| 30 | 1.0541488748344063 |
| 34 | 0.7446304570524487 |
| 36 | 0.9016343556232885 |
| 38 | 0.7563497631862197 |
| 41 | 0.47288650679876015 |
| 43 | 0.7676970417489387 |
| 44 | 1.1005842683014062 |
| 45 | 1.063859481719135 |
| 49 | 0.20933076894188388 |
| 58 | 0.7280152386717528 |
| 59 | 0.9355162171139334 |
| 60 | 0.7713168133503056 |
| 64 | 0.024166793106349393 |
| 66 | 0.9463647706764102 |
| 70 | 0.5928767674367912 |
| 79 | 0.8930804651150718 |
| 83 | 0.7954684177327636 |
| 88 | 0.7763929008367692 |
| 90 | 0.9824616725001625 |
| 93 | 0.7794469974688827 |
| 96 | 1.0615505811169912 |
| 97 | 1.0046288659848848 |
| 100 | 1.0167670074927861 |CCLP
Cell viability (relative)
**
*
**
CCLP
Cell viability (relative)
B
CCLP
EC50 24hr 1.33 mM
RBE
EC50 24hr 1.11 mM
Cell viability (%)
Cell viability (%)
Log [Niclosamide] M
Log [Niclosamide] M
KKU-M055
EC50 24hr 0.75 mM
BECs
EC50 24hr 0.82 mM
Cell viability (%)
Cell viability (%)
Log [Niclosamide] M
Log [Niclosamide] M

## Slide 2
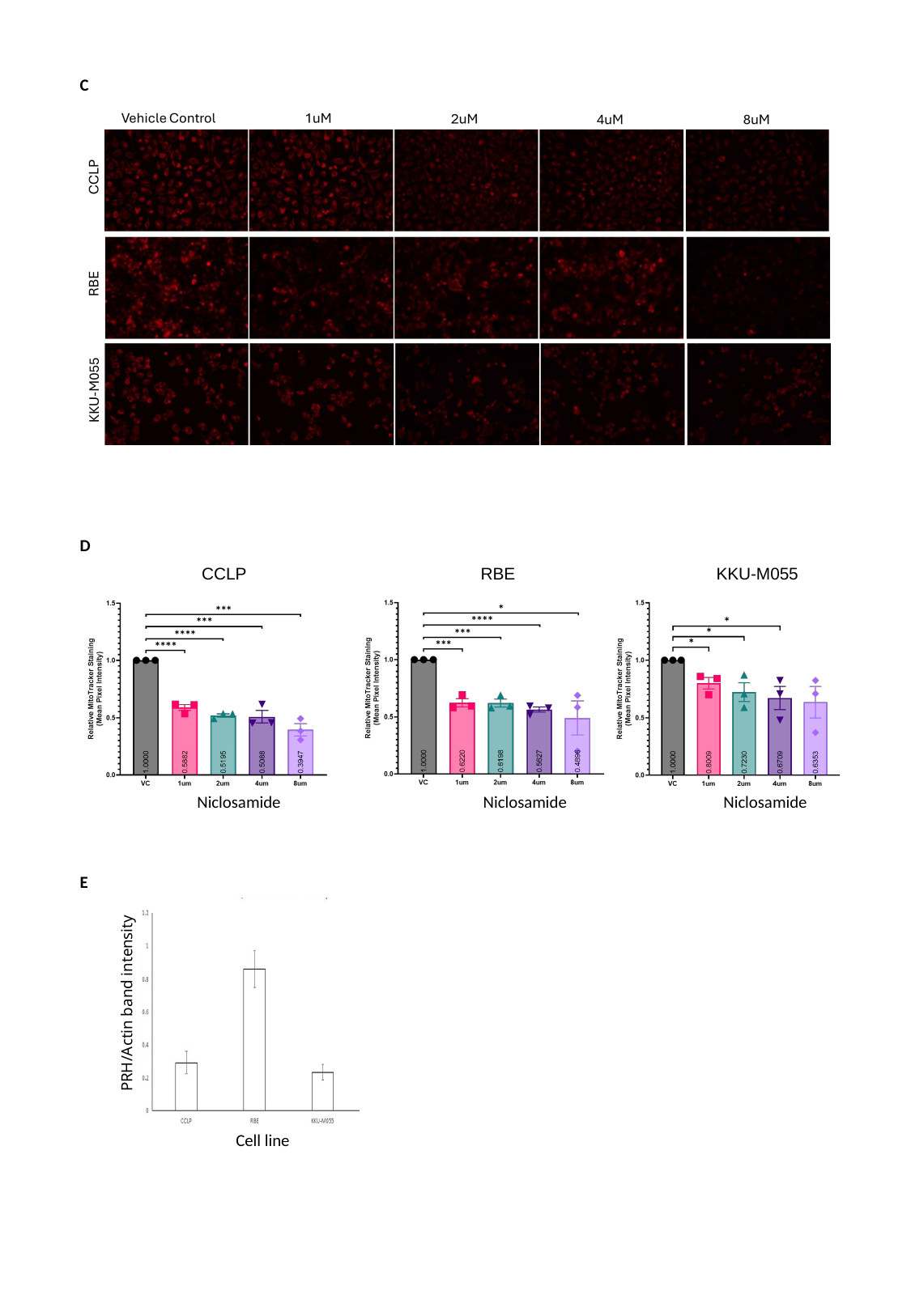

C
D
 CCLP
 RBE
 KKU-M055
 Niclosamide
 Niclosamide
 Niclosamide
E
PRH/Actin band intensity
 Cell line

## Slide 3
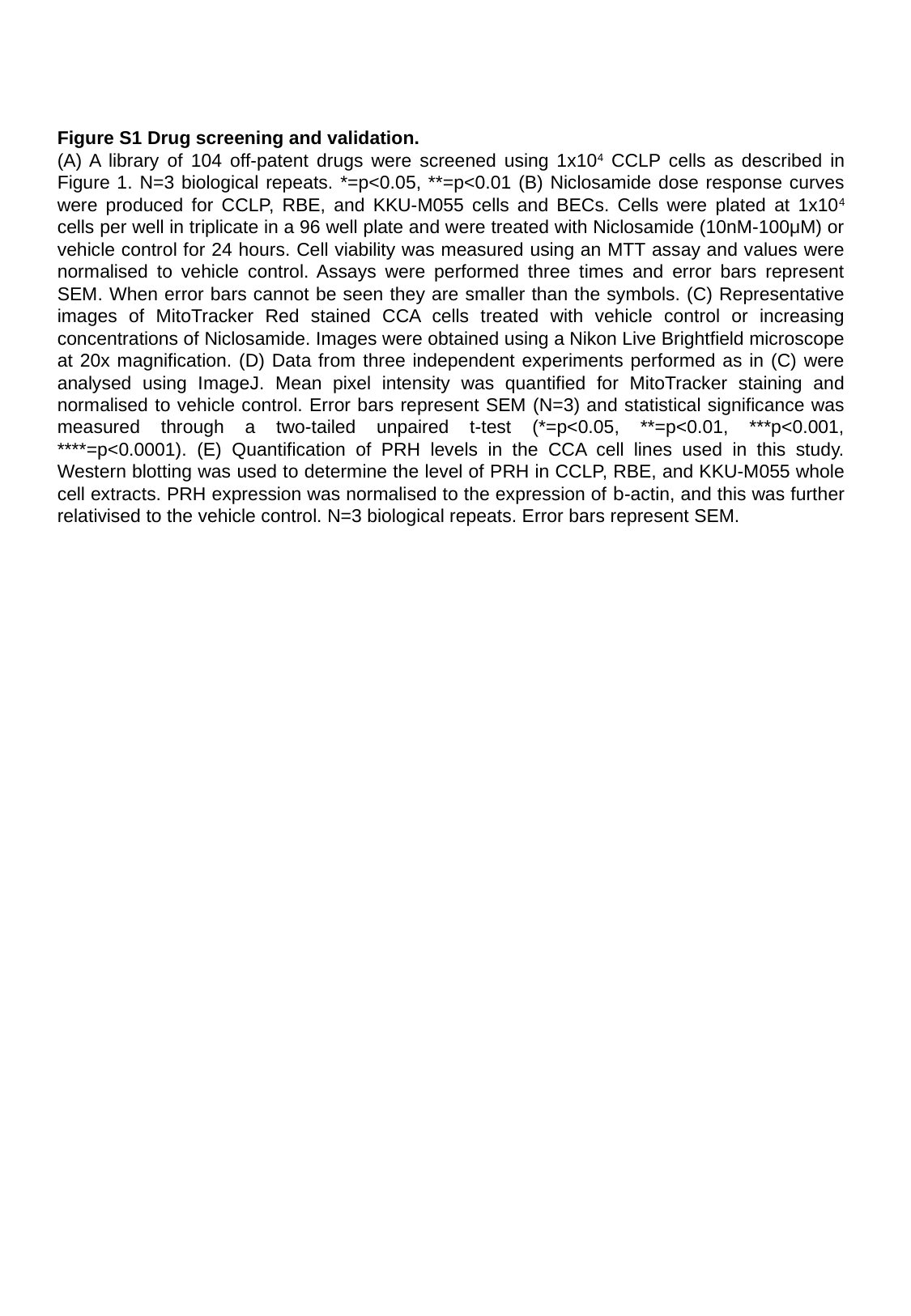

Figure S1 Drug screening and validation.
(A) A library of 104 off-patent drugs were screened using 1x10^4^ CCLP cells as described in Figure 1. N=3 biological repeats. *=p<0.05, **=p<0.01 (B) Niclosamide dose response curves were produced for CCLP, RBE, and KKU-M055 cells and BECs. Cells were plated at 1x10^4^ cells per well in triplicate in a 96 well plate and were treated with Niclosamide (10nM-100μM) or vehicle control for 24 hours. Cell viability was measured using an MTT assay and values were normalised to vehicle control. Assays were performed three times and error bars represent SEM. When error bars cannot be seen they are smaller than the symbols. (C) Representative images of MitoTracker Red stained CCA cells treated with vehicle control or increasing concentrations of Niclosamide. Images were obtained using a Nikon Live Brightfield microscope at 20x magnification. (D) Data from three independent experiments performed as in (C) were analysed using ImageJ. Mean pixel intensity was quantified for MitoTracker staining and normalised to vehicle control. Error bars represent SEM (N=3) and statistical significance was measured through a two-tailed unpaired t-test (*=p<0.05, **=p<0.01, ***p<0.001, ****=p<0.0001). (E) Quantification of PRH levels in the CCA cell lines used in this study. Western blotting was used to determine the level of PRH in CCLP, RBE, and KKU-M055 whole cell extracts. PRH expression was normalised to the expression of b-actin, and this was further relativised to the vehicle control. N=3 biological repeats. Error bars represent SEM.

## Slide 4
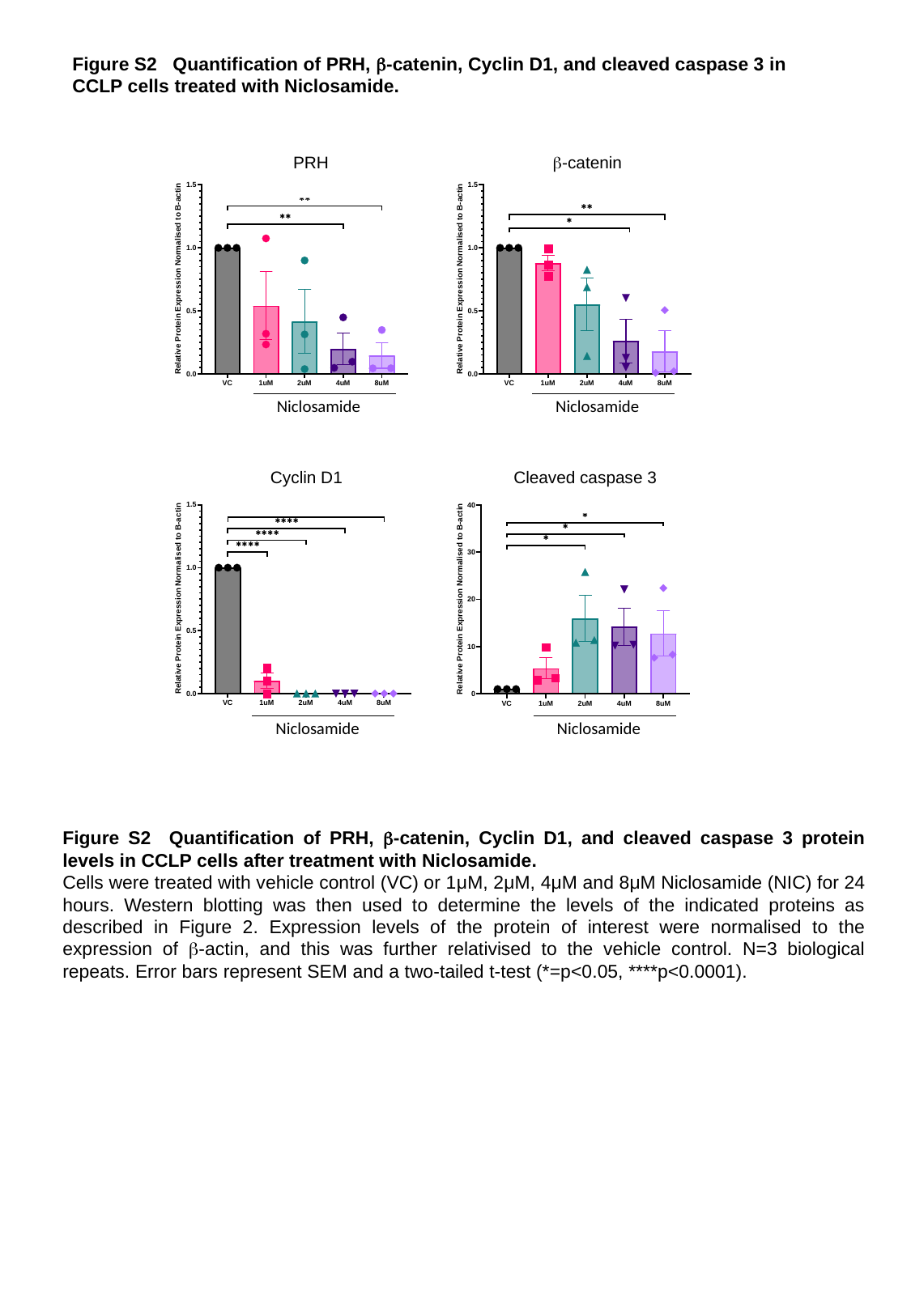

Figure S2 Quantification of PRH, b-catenin, Cyclin D1, and cleaved caspase 3 in CCLP cells treated with Niclosamide.
 PRH
 Niclosamide
 b-catenin
 Niclosamide
 Cyclin D1
 Niclosamide
Cleaved caspase 3
 Niclosamide
Figure S2 Quantification of PRH, b-catenin, Cyclin D1, and cleaved caspase 3 protein levels in CCLP cells after treatment with Niclosamide.
Cells were treated with vehicle control (VC) or 1μM, 2μM, 4μM and 8μM Niclosamide (NIC) for 24 hours. Western blotting was then used to determine the levels of the indicated proteins as described in Figure 2. Expression levels of the protein of interest were normalised to the expression of b-actin, and this was further relativised to the vehicle control. N=3 biological repeats. Error bars represent SEM and a two-tailed t-test (*=p<0.05, ****p<0.0001).

## Slide 5
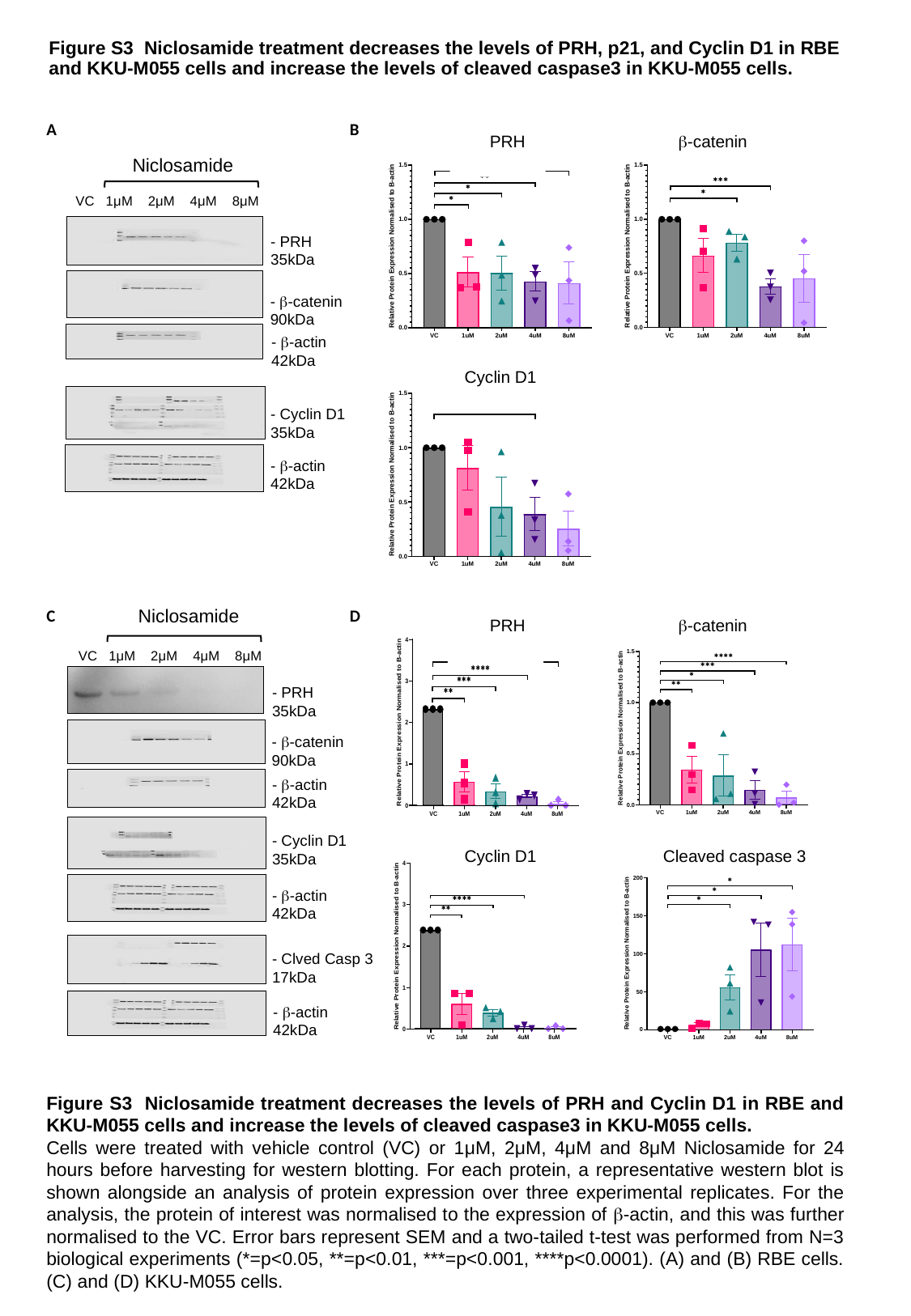

Figure S3 Niclosamide treatment decreases the levels of PRH, p21, and Cyclin D1 in RBE and KKU-M055 cells and increase the levels of cleaved caspase3 in KKU-M055 cells.
A
B
 PRH
 b-catenin
Niclosamide
VC 1μM 2μM 4μM 8μM
- PRH
35kDa
- b-catenin
90kDa
- b-actin
42kDa
- Cyclin D1
35kDa
- b-actin
42kDa
 Cyclin D1
Niclosamide
C
D
 PRH
 b-catenin
 PRH
VC 1μM 2μM 4μM 8μM
- PRH
35kDa
- b-catenin
90kDa
- b-actin
42kDa
- Cyclin D1
35kDa
 Cyclin D1
 Cleaved caspase 3
- b-actin
42kDa
- Clved Casp 3
17kDa
- b-actin
42kDa
Figure S3 Niclosamide treatment decreases the levels of PRH and Cyclin D1 in RBE and KKU-M055 cells and increase the levels of cleaved caspase3 in KKU-M055 cells.
Cells were treated with vehicle control (VC) or 1μM, 2μM, 4μM and 8μM Niclosamide for 24 hours before harvesting for western blotting. For each protein, a representative western blot is shown alongside an analysis of protein expression over three experimental replicates. For the analysis, the protein of interest was normalised to the expression of b-actin, and this was further normalised to the VC. Error bars represent SEM and a two-tailed t-test was performed from N=3 biological experiments (*=p<0.05, **=p<0.01, ***=p<0.001, ****p<0.0001). (A) and (B) RBE cells. (C) and (D) KKU-M055 cells.

## Slide 6
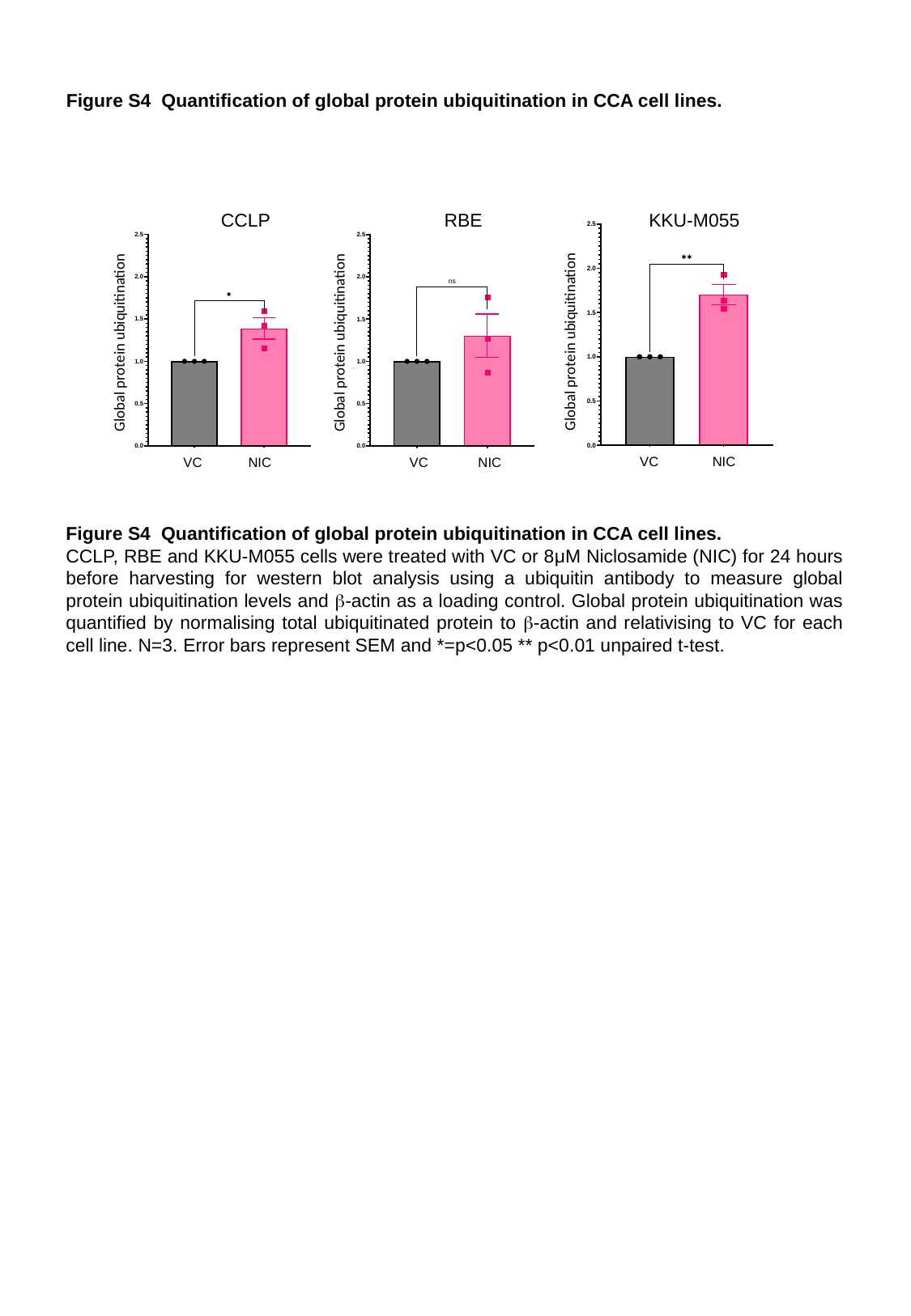

# Figure S4 Quantification of global protein ubiquitination in CCA cell lines.
CCLP
RBE
KKU-M055
Global protein ubiquitination
Global protein ubiquitination
Global protein ubiquitination
 VC NIC
VC NIC
 VC NIC
Figure S4 Quantification of global protein ubiquitination in CCA cell lines.
CCLP, RBE and KKU-M055 cells were treated with VC or 8μM Niclosamide (NIC) for 24 hours before harvesting for western blot analysis using a ubiquitin antibody to measure global protein ubiquitination levels and b-actin as a loading control. Global protein ubiquitination was quantified by normalising total ubiquitinated protein to b-actin and relativising to VC for each cell line. N=3. Error bars represent SEM and *=p<0.05 ** p<0.01 unpaired t-test.

## Slide 7
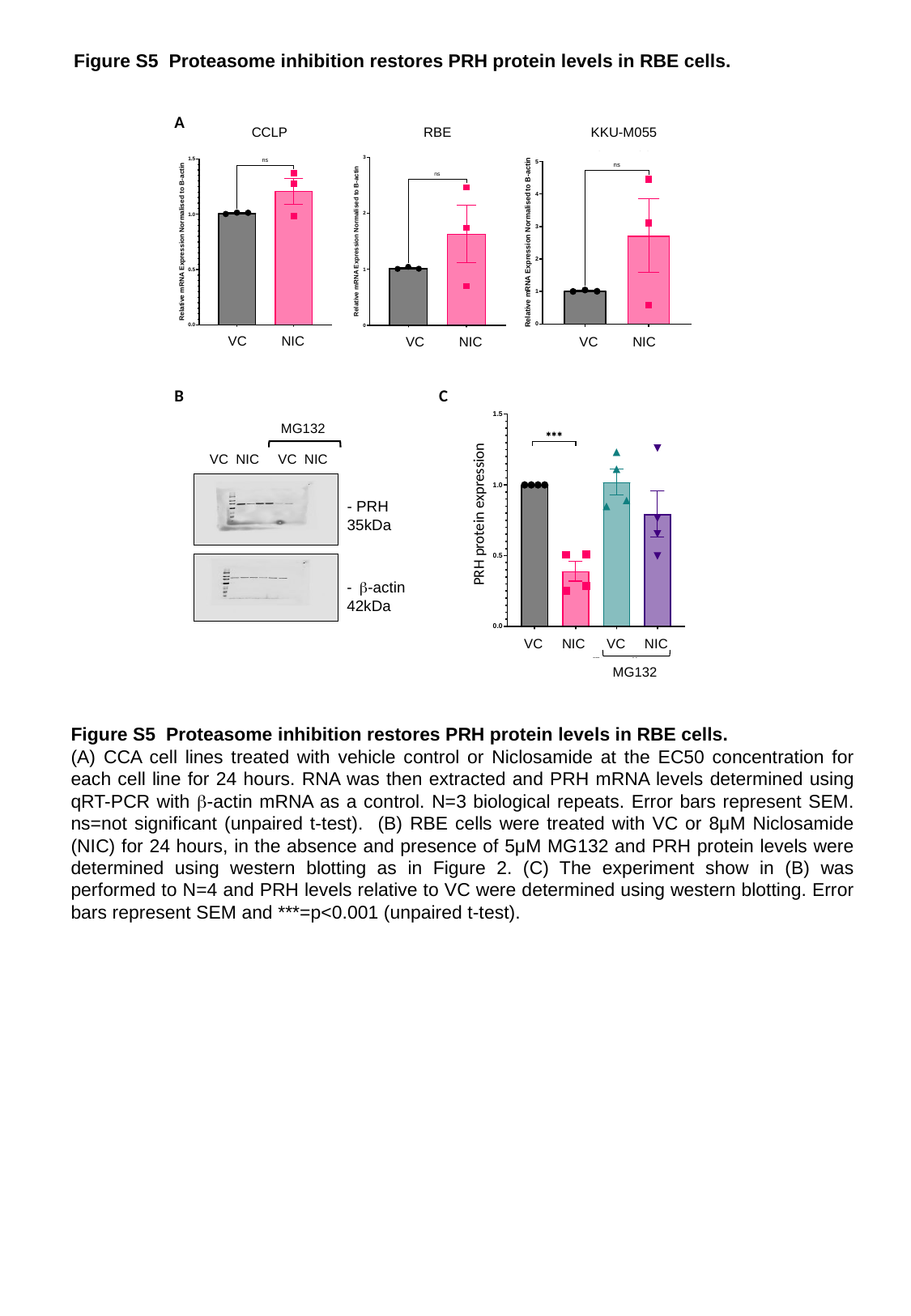

Figure S5 Proteasome inhibition restores PRH protein levels in RBE cells.
A
CCLP
RBE
KKU-M055
VC NIC
VC NIC
VC NIC
VC NIC
 PRH protein expression
VC NIC
 VC NIC
 MG132
B
C
MG132
VC NIC
VC NIC
- PRH
35kDa
- b-actin
42kDa
Figure S5 Proteasome inhibition restores PRH protein levels in RBE cells.
(A) CCA cell lines treated with vehicle control or Niclosamide at the EC50 concentration for each cell line for 24 hours. RNA was then extracted and PRH mRNA levels determined using qRT-PCR with b-actin mRNA as a control. N=3 biological repeats. Error bars represent SEM. ns=not significant (unpaired t-test). (B) RBE cells were treated with VC or 8μM Niclosamide (NIC) for 24 hours, in the absence and presence of 5μM MG132 and PRH protein levels were determined using western blotting as in Figure 2. (C) The experiment show in (B) was performed to N=4 and PRH levels relative to VC were determined using western blotting. Error bars represent SEM and ***=p<0.001 (unpaired t-test).

## Slide 8
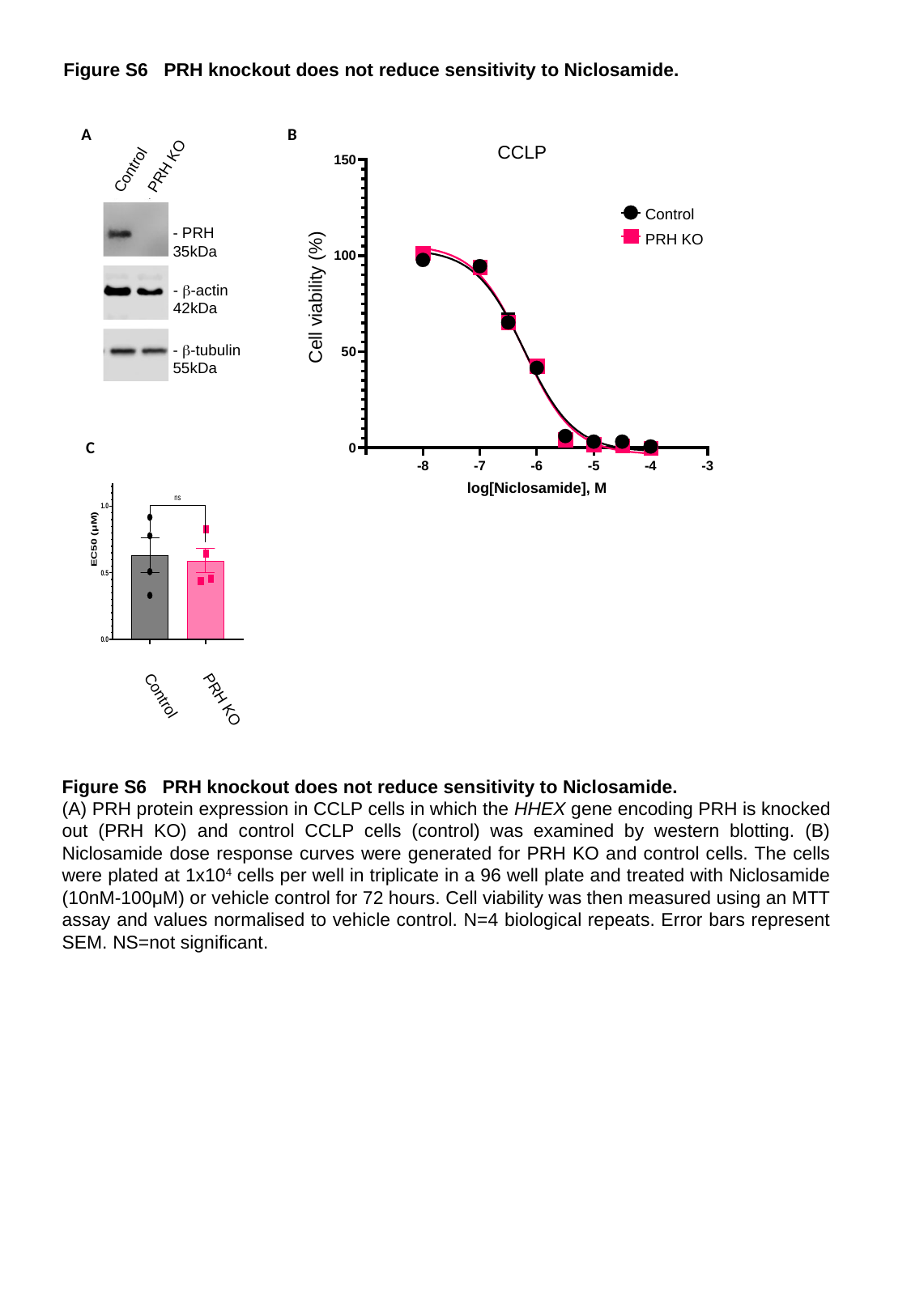

Figure S6 PRH knockout does not reduce sensitivity to Niclosamide.
Control
PRH KO
- PRH
35kDa
- b-actin
42kDa
- b-tubulin
55kDa
CCLP
Control
PRH KO
A
B
Cell viability (%)
C
D
Control
PRH KO
Figure S6 PRH knockout does not reduce sensitivity to Niclosamide.
(A) PRH protein expression in CCLP cells in which the HHEX gene encoding PRH is knocked out (PRH KO) and control CCLP cells (control) was examined by western blotting. (B) Niclosamide dose response curves were generated for PRH KO and control cells. The cells were plated at 1x10^4^ cells per well in triplicate in a 96 well plate and treated with Niclosamide (10nM-100μM) or vehicle control for 72 hours. Cell viability was then measured using an MTT assay and values normalised to vehicle control. N=4 biological repeats. Error bars represent SEM. NS=not significant.

## Slide 9
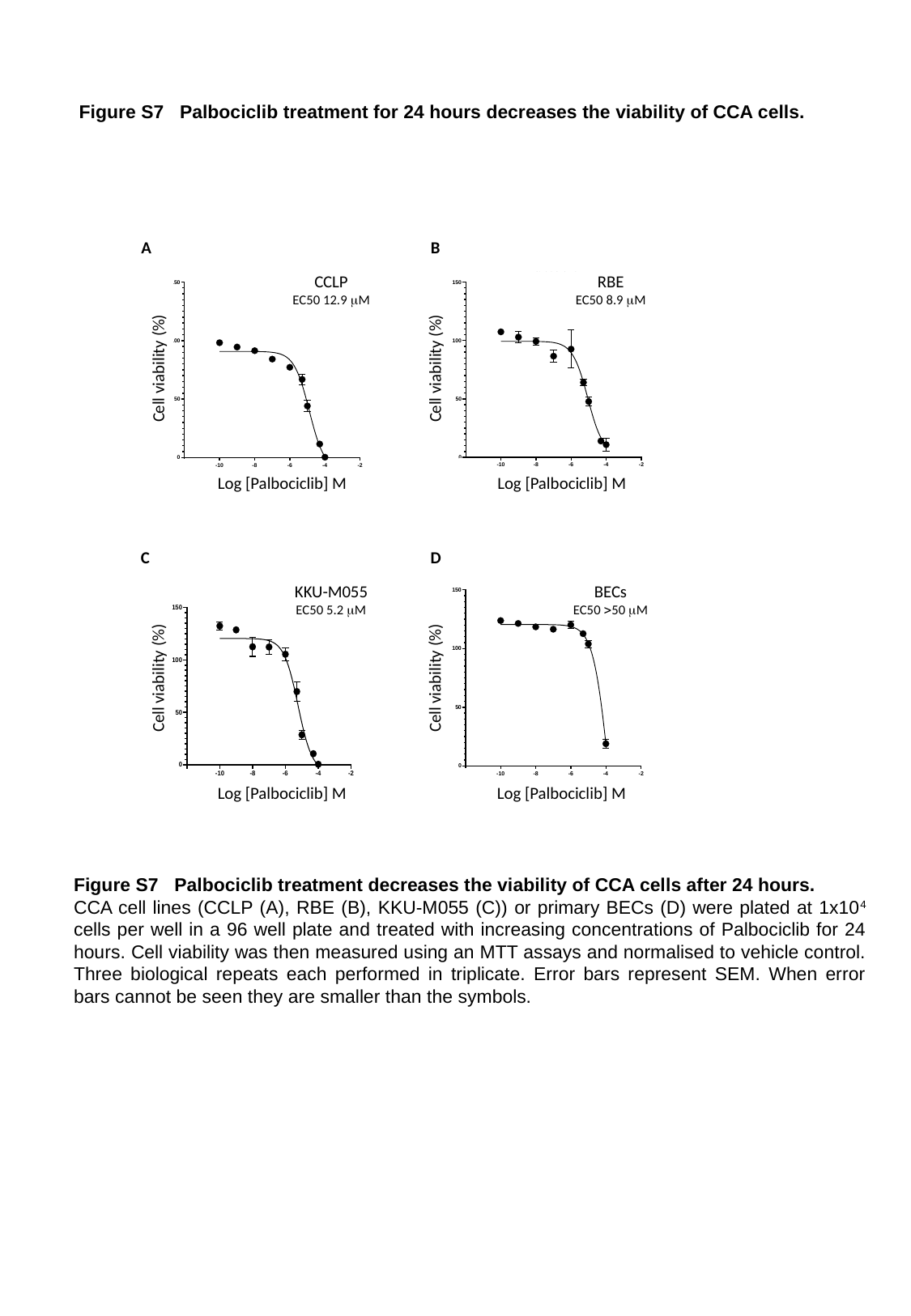

Figure S7 Palbociclib treatment for 24 hours decreases the viability of CCA cells.
A
B
CCLP
EC50 12.9 mM
RBE
EC50 8.9 mM
Cell viability (%)
Cell viability (%)
Log [Palbociclib] M
Log [Palbociclib] M
C
D
KKU-M055
EC50 5.2 mM
BECs
EC50 50 mM
Cell viability (%)
Cell viability (%)
Log [Palbociclib] M
Log [Palbociclib] M
Figure S7 Palbociclib treatment decreases the viability of CCA cells after 24 hours.
CCA cell lines (CCLP (A), RBE (B), KKU-M055 (C)) or primary BECs (D) were plated at 1x10^4^ cells per well in a 96 well plate and treated with increasing concentrations of Palbociclib for 24 hours. Cell viability was then measured using an MTT assays and normalised to vehicle control. Three biological repeats each performed in triplicate. Error bars represent SEM. When error bars cannot be seen they are smaller than the symbols.

## Slide 10
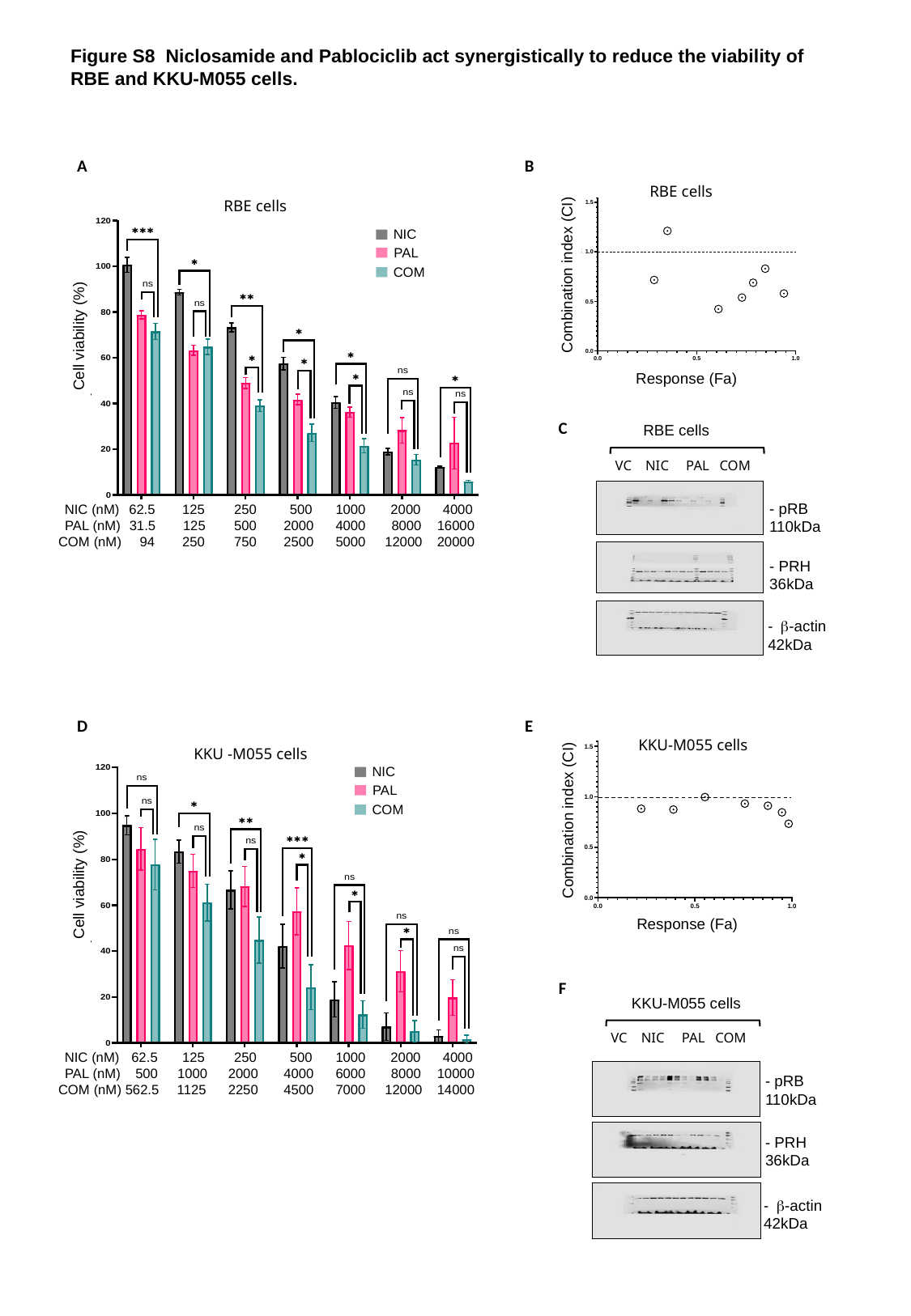

# Figure S8 Niclosamide and Pablociclib act synergistically to reduce the viability of RBE and KKU-M055 cells.
A
B
RBE cells
RBE cells
NIC
PAL
COM
Combination index (CI)
Cell viability (%)
Response (Fa)
C
RBE cells
 VC NIC PAL COM
- pRB
110kDa
- PRH
36kDa
- b-actin
42kDa
NIC (nM)
62.5
125
250
500
1000
2000
4000
PAL (nM)
31.5
125
500
2000
4000
8000
16000
COM (nM)
94
250
750
2500
5000
12000
20000
KKU -M055 cells
NIC
PAL
COM
Cell viability (%)
NIC (nM)
62.5
125
250
500
1000
2000
4000
PAL (nM)
500
1000
2000
4000
6000
8000
10000
COM (nM)
562.5
1125
2250
4500
7000
12000
14000
D
E
KKU-M055 cells
Combination index (CI)
Response (Fa)
F
KKU-M055 cells
 VC NIC PAL COM
- pRB
110kDa
- PRH
36kDa
- b-actin
42kDa

## Slide 11
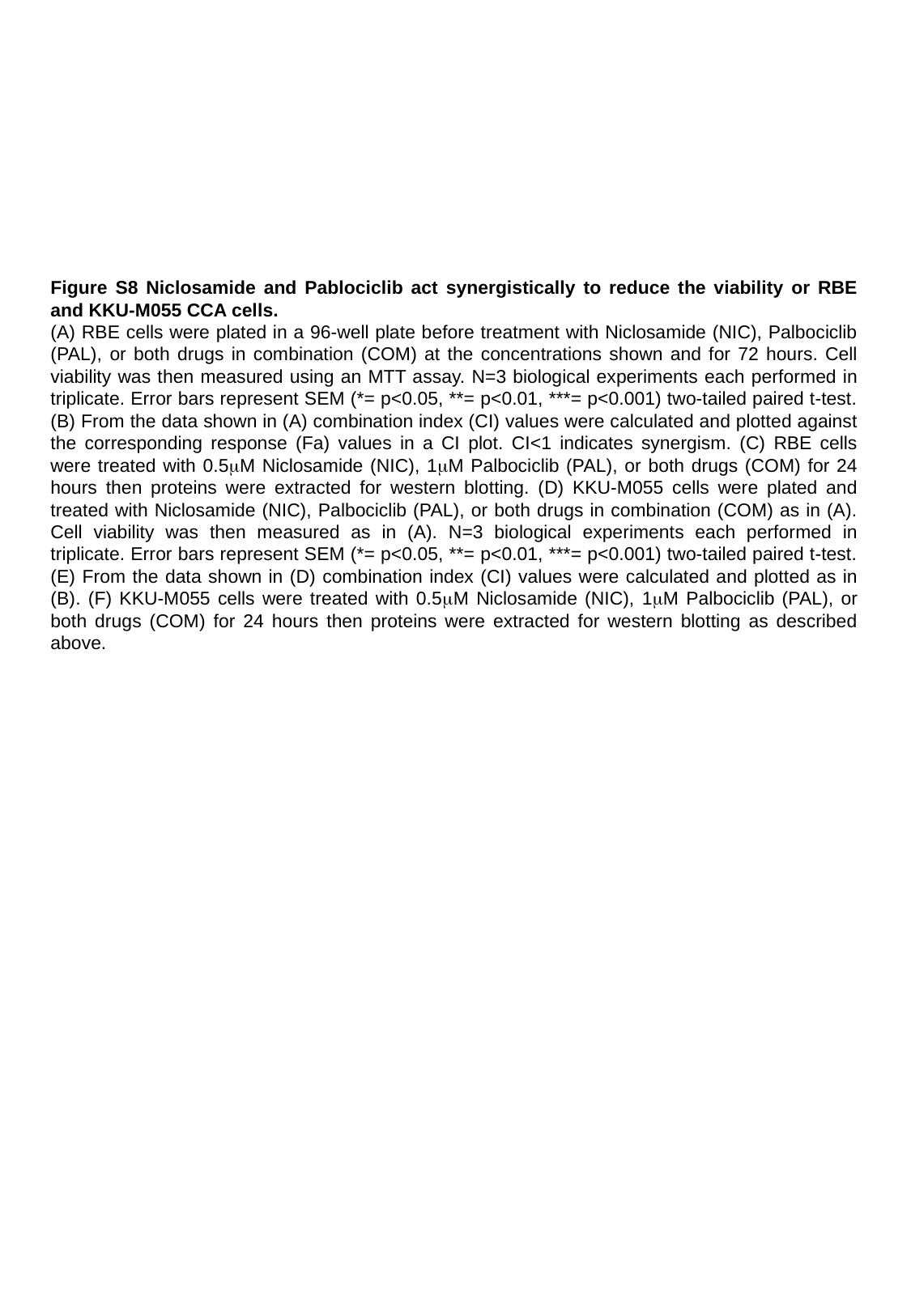

Figure S8 Niclosamide and Pablociclib act synergistically to reduce the viability or RBE and KKU-M055 CCA cells.
(A) RBE cells were plated in a 96-well plate before treatment with Niclosamide (NIC), Palbociclib (PAL), or both drugs in combination (COM) at the concentrations shown and for 72 hours. Cell viability was then measured using an MTT assay. N=3 biological experiments each performed in triplicate. Error bars represent SEM (*= p<0.05, **= p<0.01, ***= p<0.001) two-tailed paired t-test. (B) From the data shown in (A) combination index (CI) values were calculated and plotted against the corresponding response (Fa) values in a CI plot. CI<1 indicates synergism. (C) RBE cells were treated with 0.5mM Niclosamide (NIC), 1mM Palbociclib (PAL), or both drugs (COM) for 24 hours then proteins were extracted for western blotting. (D) KKU-M055 cells were plated and treated with Niclosamide (NIC), Palbociclib (PAL), or both drugs in combination (COM) as in (A). Cell viability was then measured as in (A). N=3 biological experiments each performed in triplicate. Error bars represent SEM (*= p<0.05, **= p<0.01, ***= p<0.001) two-tailed paired t-test. (E) From the data shown in (D) combination index (CI) values were calculated and plotted as in (B). (F) KKU-M055 cells were treated with 0.5mM Niclosamide (NIC), 1mM Palbociclib (PAL), or both drugs (COM) for 24 hours then proteins were extracted for western blotting as described above.

## Slide 12
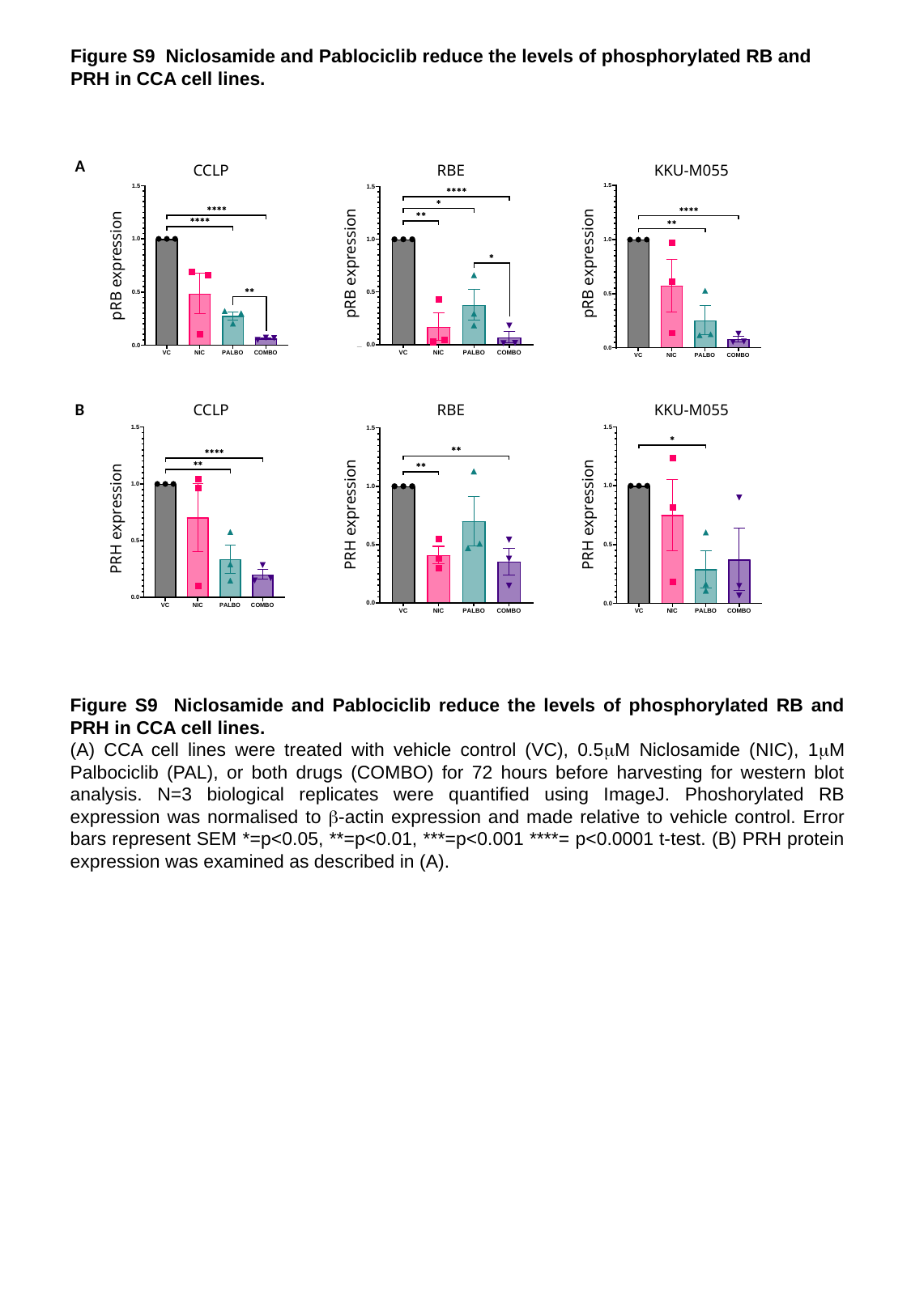

# Figure S9 Niclosamide and Pablociclib reduce the levels of phosphorylated RB and PRH in CCA cell lines.
A
 CCLP
 RBE
 KKU-M055
 pRB expression
 pRB expression
 pRB expression
B
 CCLP
 RBE
 KKU-M055
 PRH expression
 PRH expression
 PRH expression
Figure S9 Niclosamide and Pablociclib reduce the levels of phosphorylated RB and PRH in CCA cell lines.
(A) CCA cell lines were treated with vehicle control (VC), 0.5mM Niclosamide (NIC), 1mM Palbociclib (PAL), or both drugs (COMBO) for 72 hours before harvesting for western blot analysis. N=3 biological replicates were quantified using ImageJ. Phoshorylated RB expression was normalised to b-actin expression and made relative to vehicle control. Error bars represent SEM *=p<0.05, **=p<0.01, ***=p<0.001 ****= p<0.0001 t-test. (B) PRH protein expression was examined as described in (A).

## Slide 13
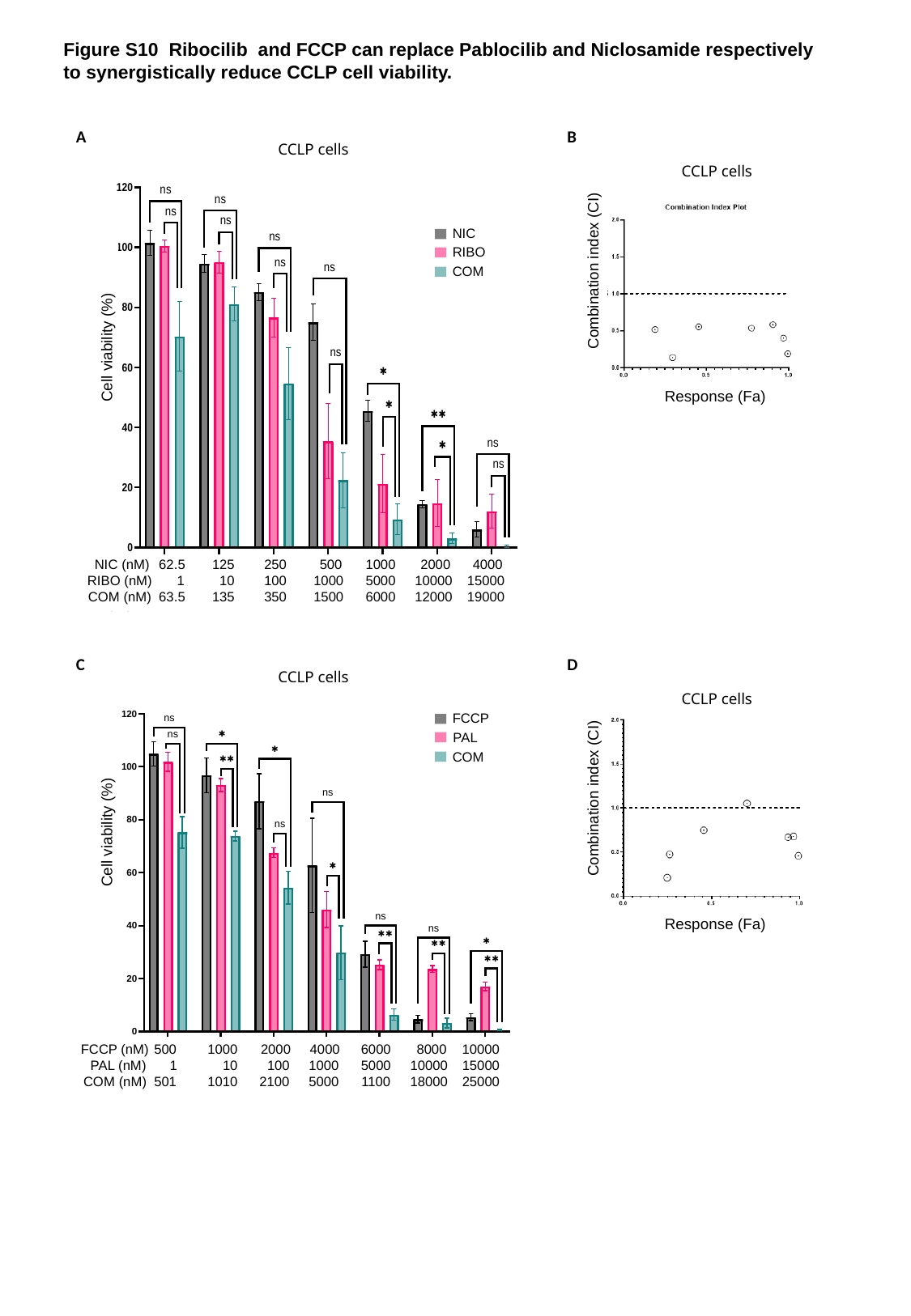

# Figure S10 Ribocilib and FCCP can replace Pablocilib and Niclosamide respectively to synergistically reduce CCLP cell viability.
A
B
CCLP cells
 CCLP cells
NIC
RIBO
COM
Combination index (CI)
Cell viability (%)
Response (Fa)
NIC (nM)
62.5
125
250
500
1000
2000
4000
RIBO (nM)
1
10
100
1000
5000
10000
15000
COM (nM)
63.5
135
350
1500
6000
12000
19000
C
D
CCLP cells
FCCP
PAL
COM
Cell viability (%)
FCCP (nM)
500
1000
2000
4000
6000
8000
10000
PAL (nM)
1
10
100
1000
5000
10000
15000
COM (nM)
501
1010
2100
5000
1100
18000
25000
 CCLP cells
Combination index (CI)
Response (Fa)

## Slide 14
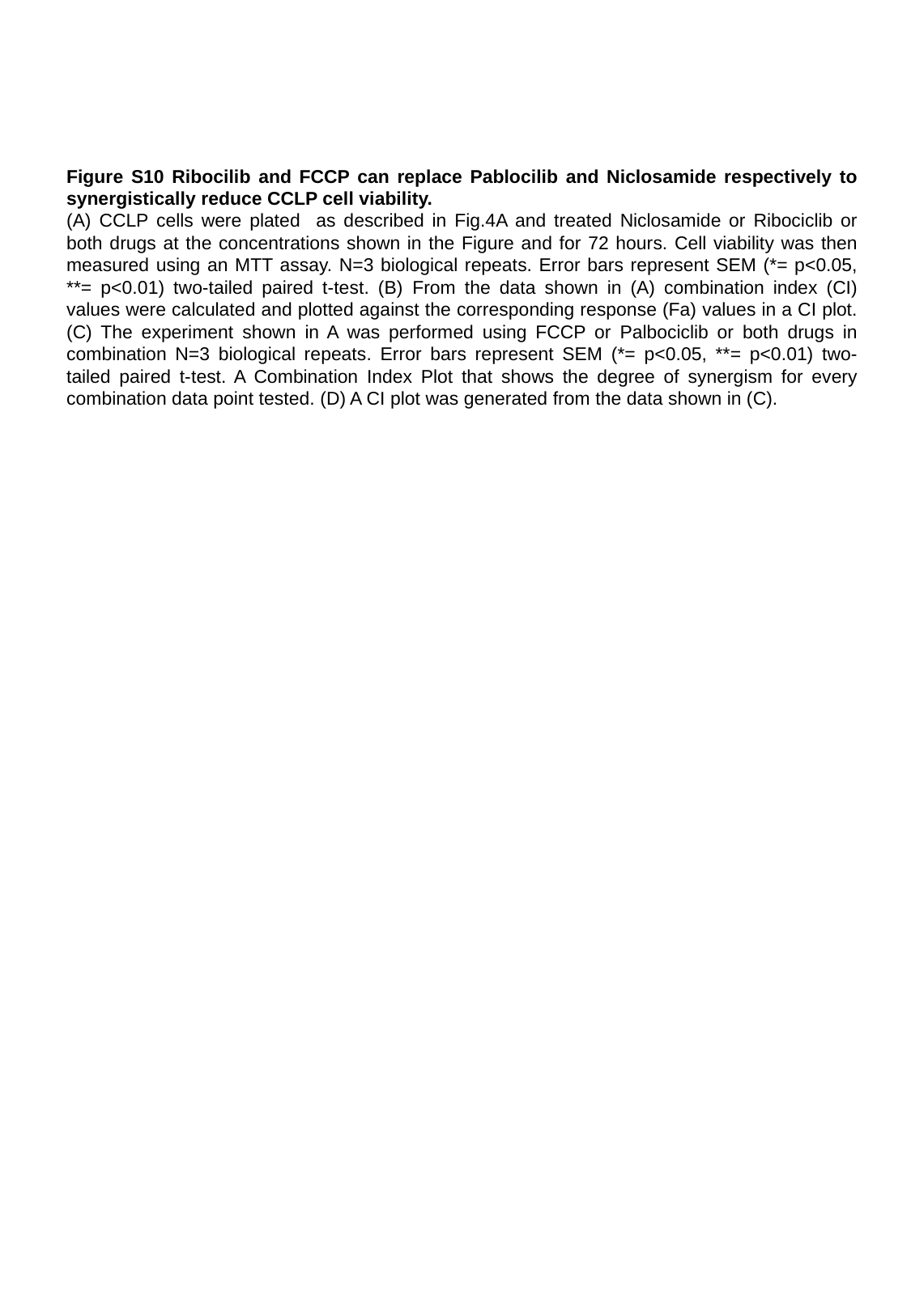

Figure S10 Ribocilib and FCCP can replace Pablocilib and Niclosamide respectively to synergistically reduce CCLP cell viability.
(A) CCLP cells were plated as described in Fig.4A and treated Niclosamide or Ribociclib or both drugs at the concentrations shown in the Figure and for 72 hours. Cell viability was then measured using an MTT assay. N=3 biological repeats. Error bars represent SEM (*= p<0.05, **= p<0.01) two-tailed paired t-test. (B) From the data shown in (A) combination index (CI) values were calculated and plotted against the corresponding response (Fa) values in a CI plot. (C) The experiment shown in A was performed using FCCP or Palbociclib or both drugs in combination N=3 biological repeats. Error bars represent SEM (*= p<0.05, **= p<0.01) two-tailed paired t-test. A Combination Index Plot that shows the degree of synergism for every combination data point tested. (D) A CI plot was generated from the data shown in (C).

## Slide 15
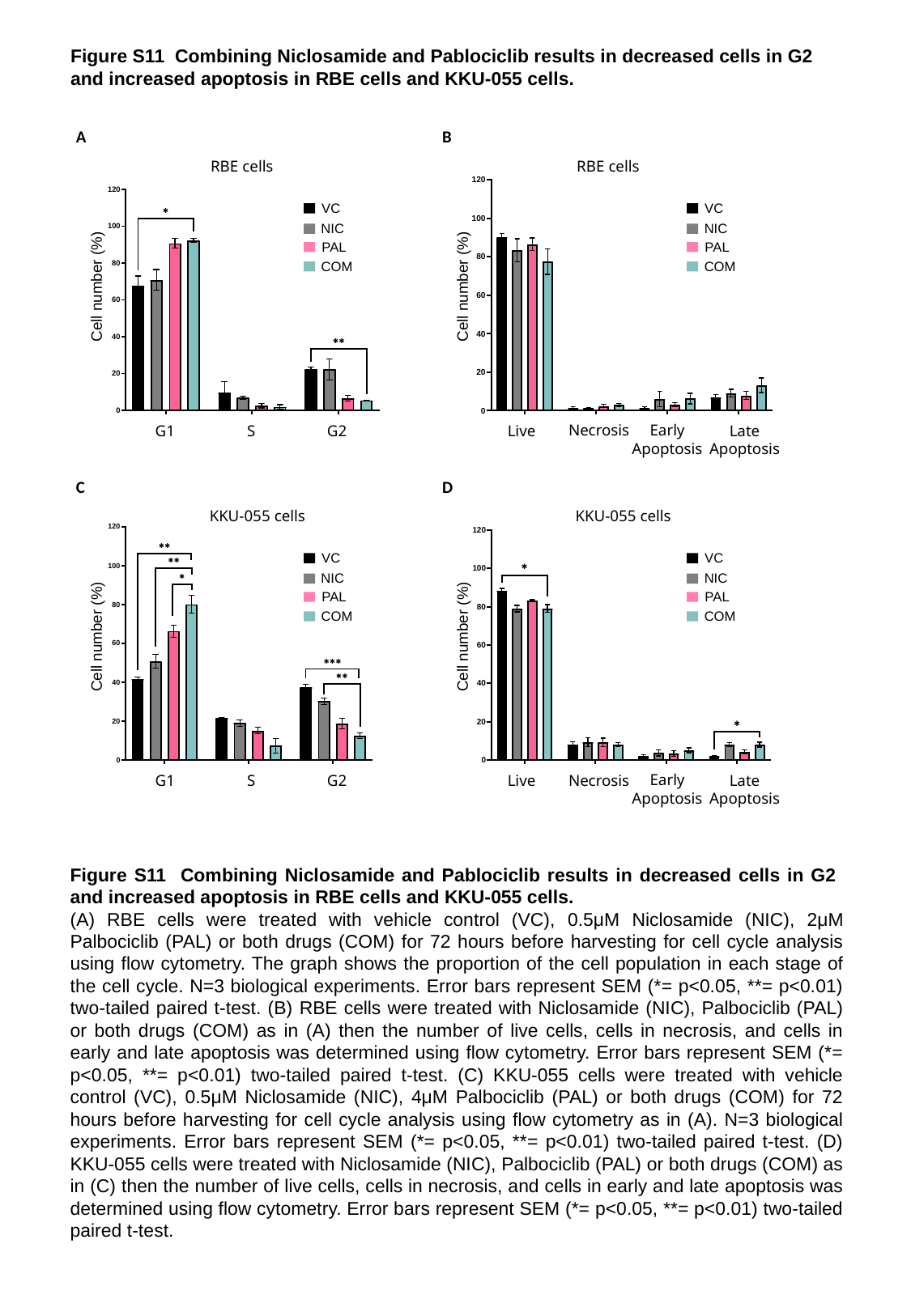

# Figure S11 Combining Niclosamide and Pablociclib results in decreased cells in G2 and increased apoptosis in RBE cells and KKU-055 cells.
A
B
RBE cells
RBE cells
VC
NIC
PAL
COM
VC
NIC
PAL
COM
Cell number (%)
Cell number (%)
Necrosis
 Early
Apoptosis
G1
S
G2
Live
Late
Apoptosis
C
D
KKU-055 cells
KKU-055 cells
VC
NIC
PAL
COM
VC
NIC
PAL
COM
Cell number (%)
Cell number (%)
Necrosis
 Early
Apoptosis
G1
S
G2
Live
Late
Apoptosis
Figure S11 Combining Niclosamide and Pablociclib results in decreased cells in G2 and increased apoptosis in RBE cells and KKU-055 cells.
(A) RBE cells were treated with vehicle control (VC), 0.5μM Niclosamide (NIC), 2μM Palbociclib (PAL) or both drugs (COM) for 72 hours before harvesting for cell cycle analysis using flow cytometry. The graph shows the proportion of the cell population in each stage of the cell cycle. N=3 biological experiments. Error bars represent SEM (*= p<0.05, **= p<0.01) two-tailed paired t-test. (B) RBE cells were treated with Niclosamide (NIC), Palbociclib (PAL) or both drugs (COM) as in (A) then the number of live cells, cells in necrosis, and cells in early and late apoptosis was determined using flow cytometry. Error bars represent SEM (*= p<0.05, **= p<0.01) two-tailed paired t-test. (C) KKU-055 cells were treated with vehicle control (VC), 0.5μM Niclosamide (NIC), 4μM Palbociclib (PAL) or both drugs (COM) for 72 hours before harvesting for cell cycle analysis using flow cytometry as in (A). N=3 biological experiments. Error bars represent SEM (*= p<0.05, **= p<0.01) two-tailed paired t-test. (D) KKU-055 cells were treated with Niclosamide (NIC), Palbociclib (PAL) or both drugs (COM) as in (C) then the number of live cells, cells in necrosis, and cells in early and late apoptosis was determined using flow cytometry. Error bars represent SEM (*= p<0.05, **= p<0.01) two-tailed paired t-test.

## Slide 16
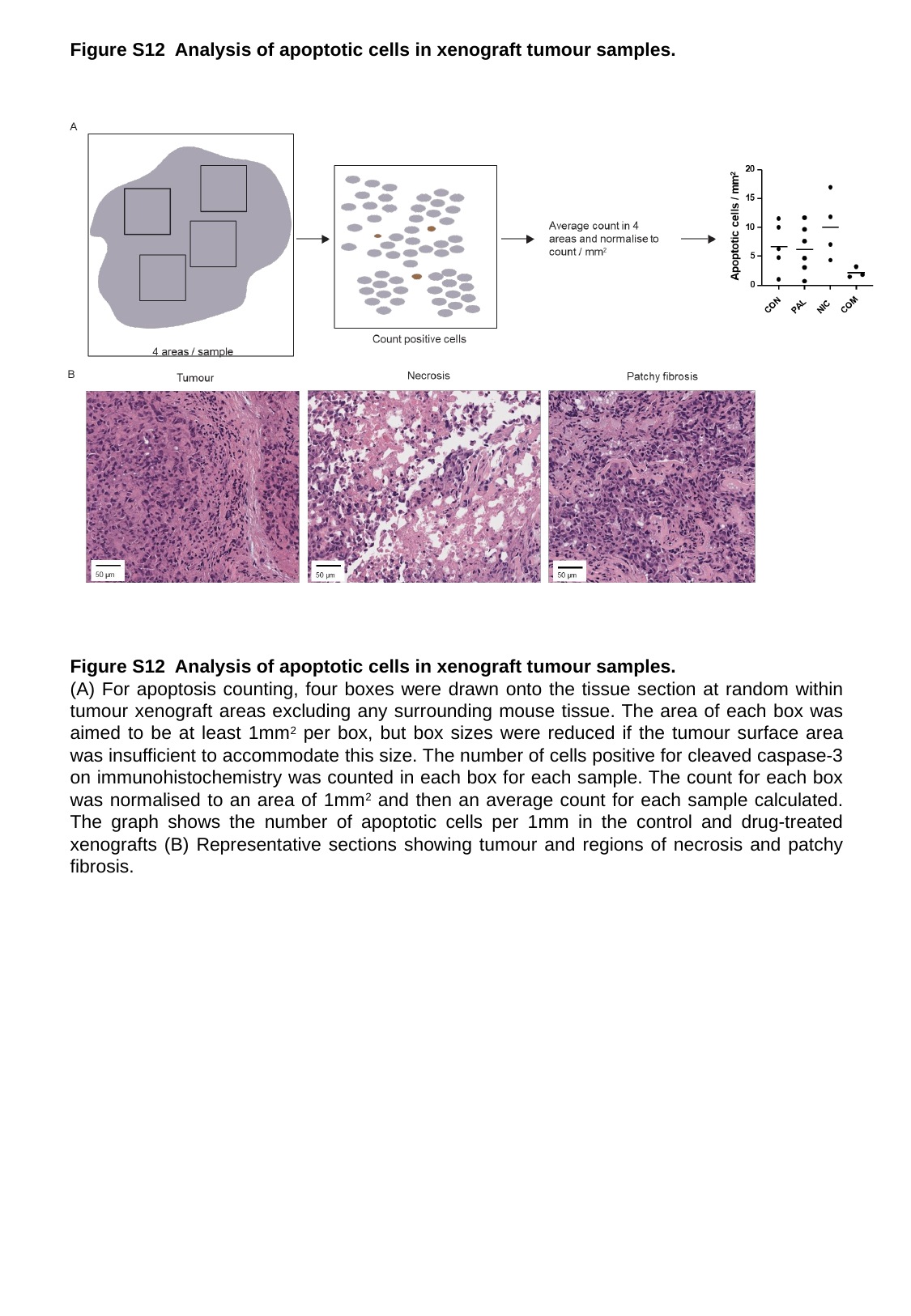

Figure S12 Analysis of apoptotic cells in xenograft tumour samples.
Figure S12 Analysis of apoptotic cells in xenograft tumour samples.
(A) For apoptosis counting, four boxes were drawn onto the tissue section at random within tumour xenograft areas excluding any surrounding mouse tissue. The area of each box was aimed to be at least 1mm2 per box, but box sizes were reduced if the tumour surface area was insufficient to accommodate this size. The number of cells positive for cleaved caspase-3 on immunohistochemistry was counted in each box for each sample. The count for each box was normalised to an area of 1mm2 and then an average count for each sample calculated. The graph shows the number of apoptotic cells per 1mm in the control and drug-treated xenografts (B) Representative sections showing tumour and regions of necrosis and patchy fibrosis.

## Slide 17
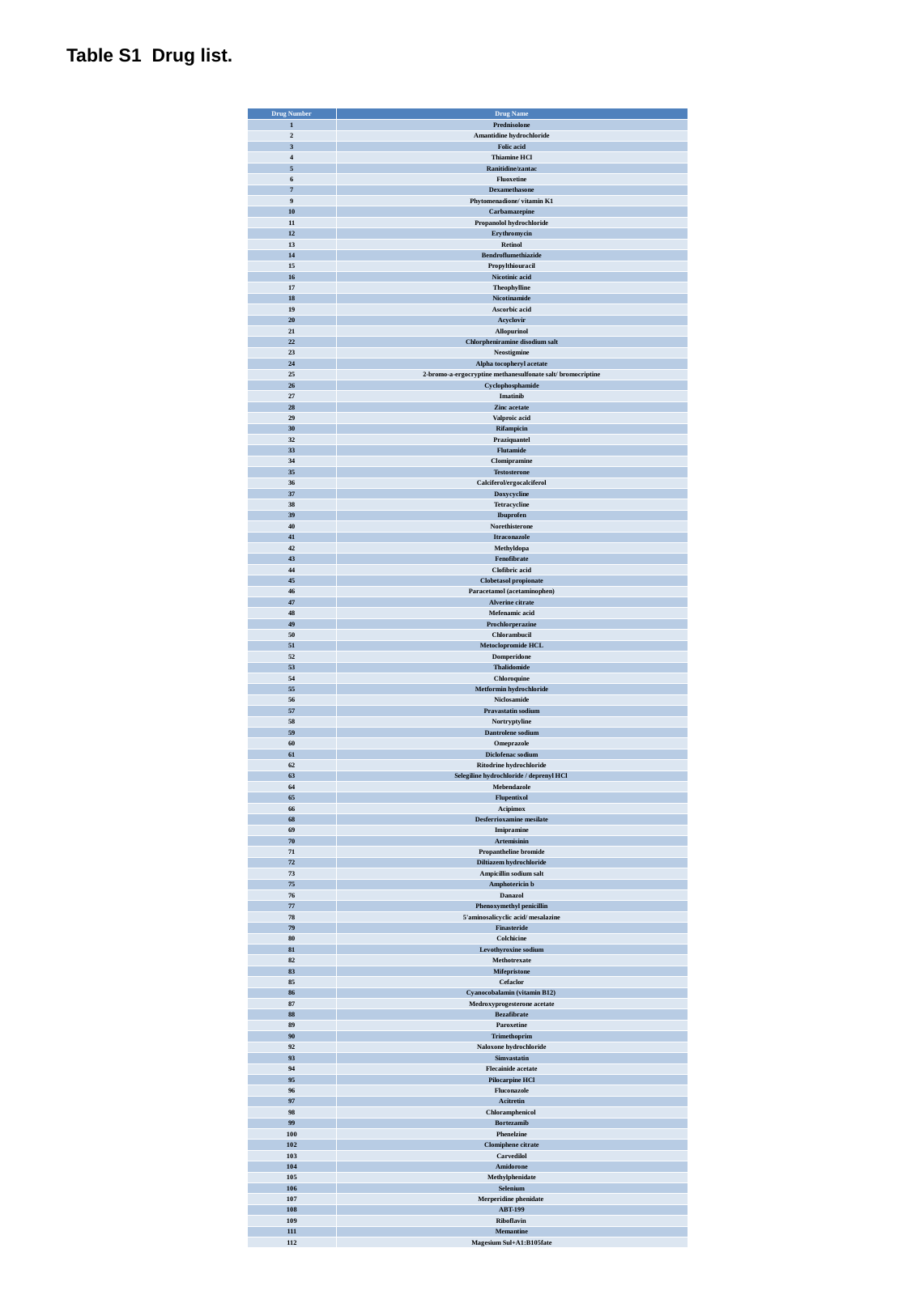

Table S1 Drug list.
| Drug Number | Drug Name |
| --- | --- |
| 1 | Prednisolone |
| 2 | Amantidine hydrochloride |
| 3 | Folic acid |
| 4 | Thiamine HCl |
| 5 | Ranitidine/zantac |
| 6 | Fluoxetine |
| 7 | Dexamethasone |
| 9 | Phytomenadione/ vitamin K1 |
| 10 | Carbamazepine |
| 11 | Propanolol hydrochloride |
| 12 | Erythromycin |
| 13 | Retinol |
| 14 | Bendroflumethiazide |
| 15 | Propylthiouracil |
| 16 | Nicotinic acid |
| 17 | Theophylline |
| 18 | Nicotinamide |
| 19 | Ascorbic acid |
| 20 | Acyclovir |
| 21 | Allopurinol |
| 22 | Chlorpheniramine disodium salt |
| 23 | Neostigmine |
| 24 | Alpha tocopheryl acetate |
| 25 | 2-bromo-a-ergocryptine methanesulfonate salt/ bromocriptine |
| 26 | Cyclophosphamide |
| 27 | Imatinib |
| 28 | Zinc acetate |
| 29 | Valproic acid |
| 30 | Rifampicin |
| 32 | Praziquantel |
| 33 | Flutamide |
| 34 | Clomipramine |
| 35 | Testosterone |
| 36 | Calciferol/ergocalciferol |
| 37 | Doxycycline |
| 38 | Tetracycline |
| 39 | Ibuprofen |
| 40 | Norethisterone |
| 41 | Itraconazole |
| 42 | Methyldopa |
| 43 | Fenofibrate |
| 44 | Clofibric acid |
| 45 | Clobetasol propionate |
| 46 | Paracetamol (acetaminophen) |
| 47 | Alverine citrate |
| 48 | Mefenamic acid |
| 49 | Prochlorperazine |
| 50 | Chlorambucil |
| 51 | Metoclopromide HCL |
| 52 | Domperidone |
| 53 | Thalidomide |
| 54 | Chloroquine |
| 55 | Metformin hydrochloride |
| 56 | Niclosamide |
| 57 | Pravastatin sodium |
| 58 | Nortryptyline |
| 59 | Dantrolene sodium |
| 60 | Omeprazole |
| 61 | Diclofenac sodium |
| 62 | Ritodrine hydrochloride |
| 63 | Selegiline hydrochloride / deprenyl HCl |
| 64 | Mebendazole |
| 65 | Flupentixol |
| 66 | Acipimox |
| 68 | Desferrioxamine mesilate |
| 69 | Imipramine |
| 70 | Artemisinin |
| 71 | Propantheline bromide |
| 72 | Diltiazem hydrochloride |
| 73 | Ampicillin sodium salt |
| 75 | Amphotericin b |
| 76 | Danazol |
| 77 | Phenoxymethyl penicillin |
| 78 | 5'aminosalicyclic acid/ mesalazine |
| 79 | Finasteride |
| 80 | Colchicine |
| 81 | Levothyroxine sodium |
| 82 | Methotrexate |
| 83 | Mifepristone |
| 85 | Cefaclor |
| 86 | Cyanocobalamin (vitamin B12) |
| 87 | Medroxyprogesterone acetate |
| 88 | Bezafibrate |
| 89 | Paroxetine |
| 90 | Trimethoprim |
| 92 | Naloxone hydrochloride |
| 93 | Simvastatin |
| 94 | Flecainide acetate |
| 95 | Pilocarpine HCl |
| 96 | Fluconazole |
| 97 | Acitretin |
| 98 | Chloramphenicol |
| 99 | Bortezamib |
| 100 | Phenelzine |
| 102 | Clomiphene citrate |
| 103 | Carvedilol |
| 104 | Amidorone |
| 105 | Methylphenidate |
| 106 | Selenium |
| 107 | Merperidine phenidate |
| 108 | ABT-199 |
| 109 | Riboflavin |
| 111 | Memantine |
| 112 | Magesium Sul+A1:B105fate |

## Slide 18
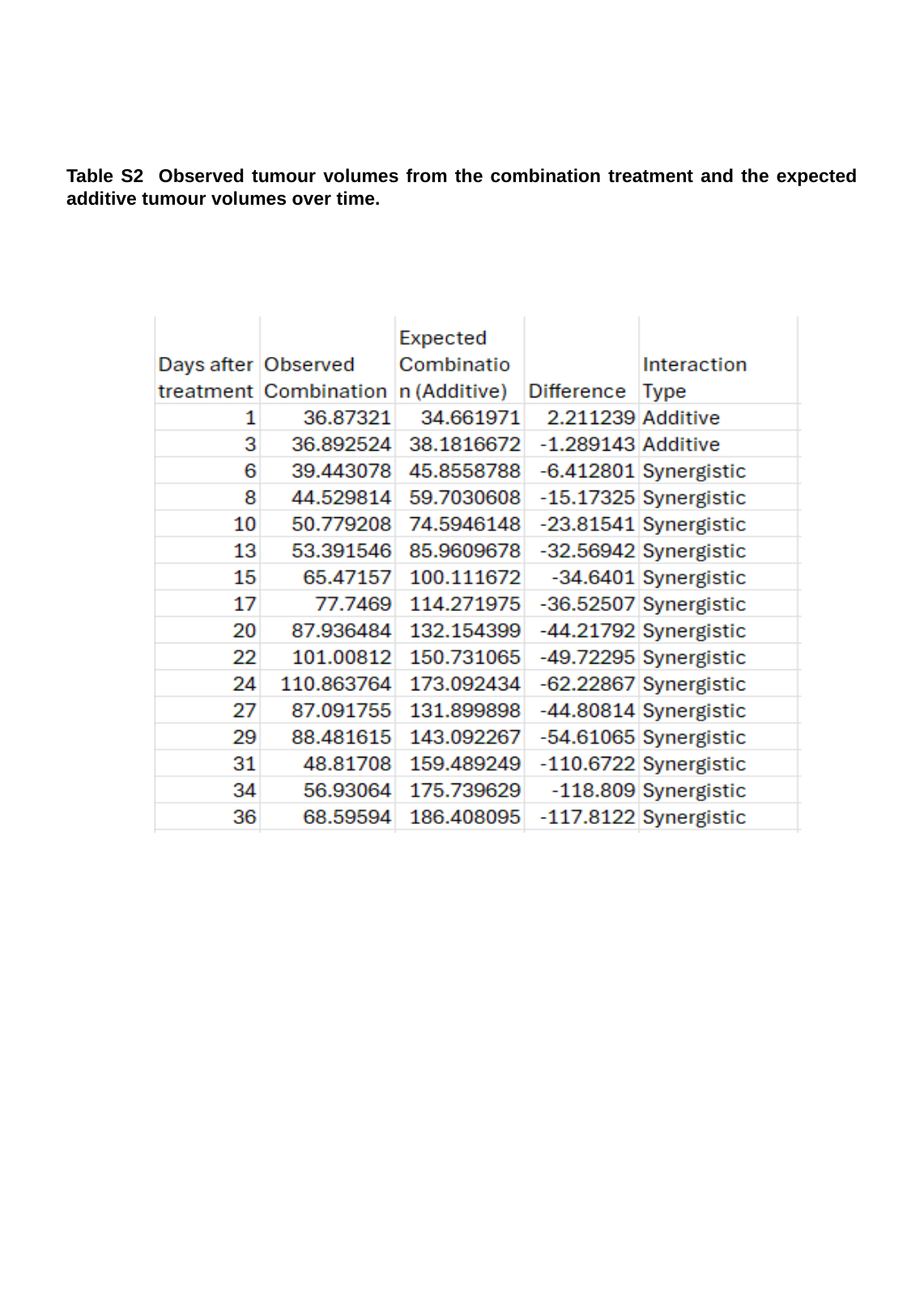

Table S2 Observed tumour volumes from the combination treatment and the expected additive tumour volumes over time.
